# Supplementary material for: Effects of a waiting list control design on alcohol consumption among online help-seekers: protocol for a randomised controlled trial
Source: BMJ Open. 2021 Aug 26;11(8):e049810. doi: 10.1136/bmjopen-2021-049810 (PMC8395291; doi:10.1136/bmjopen-2021-049810)
Supplement: Supplementary data [file bmjopen-2021-049810supp001.pdf]

APPENDIX A – INFORMED CONSENT MATERIALS

When clicking on the online advert, participants will be shown the following information about the study.

Do you want to participate in the "Digital support for alcohol" study?

Thank you for showing interest in this study. By pressing the button "I consent to take part in this study" you are consenting to take part in the study described below.

Why are we doing this study and why do you want me to participate? We have developed a digital support tool which is tailored to individual responses to a questionnaire. The tool has been designed for people who are concerned about their drinking. In order to evaluate if the tool is effective, we are asking people who search online to be part of this study.

This research project is conducted at Linköping university in Sweden, please find contact details for the primary investigator below.

What is expected of me? If you take part in the study you will first be asked to respond to a 5 minute questionnaire. Thereafter you will be asked to leave an email address to which we can send more information about the study. You will be randomly assigned to either get direct access to the tool or wait one month before being given access. One month after having signed up for the study we will send an email to you with a link to a short follow-up questionnaire.

Will I be taking any risks by participating in this study? Participating in this study will give you access to a novel digital support tool which has been designed to help people concerned about their drinking. However, you may find that the support given to you is inadequate and does not help you, and you may be de-motivated by this. You should be aware of this before deciding to take part in this study. We are however not asking you to refrain from any other support which may be available to you. For instance, you may find the information available at this website helpful: Rethinking Drinking (https://www.rethinkingdrinking.niaaa.nih.gov/).

Will I be leaving any personal information? We will collect data from you about your age, biological sex, alcohol consumption, and your readiness to change. We will also collect an email address from you so that we can contact you for follow-up one month after enrolling. The data collection is for scientific research, and is therefore motivated by public interest (GDPR EU 2016/679, Prop. 2017/18:298). Your data will be treated with confidentiality and we will take measures to ensure that no unauthorized access is granted.

Linköping University will be responsible for your personal data. According to EU law, you retain the right to, without cost, be given access to your personal data and request that changes be made. You can also request that your personal data be deleted. Your rights for deletion of data is however restricted if the data is deemed necessary for the study to be conducted. If you want access to your personal data you should contact the primary investigator Marcus Bendtsen (see information below). The data protection officer can be contacted at dataskyddsombud@liu.se. If you are not satisfied with how your personal data has been handled, you can make a formal complain to the Swedish Authority for Privacy Protection.

Where will data be stored and who will have access? During the study period we will store all data on a database at Linköping university in Sweden. After the study period the data will be archived securely at Linköping university and removed from the database. Access to the data will at first be restricted to the research team, but will later be made available for other researchers by request. Please note that once the study period is complete, all email addresses will be deleted and data will therefore be anonymous.

Who is responsible for the study? Linköping university is responsible for the data collected in this study. If you have any questions about your data you can contact Dr. Marcus Bendtsen, (marcus.bendtsen@liu.se), or the data protection officer (dataskyddsombudet@liu.se). If you are not satisfied with the way your personal data has been handled, you can file a complaint at the Swedish Data Protection Authority.

How can I get more information about the results from this study? All results will be published in peer-reviewed journals and conferences no later than 2022.

Participation is voluntary. You can at any point decide to not be part of the study and you will not have to explain why.

Contact details
Dr. Marcus Bendtsen, marcus.bendtsen@liu.se, 013-28 69 75
Docent in Medical Informatics
Senior Lecturer in Experimental Social Medicine and Public Health
Department of Health, Medicine and Caring Sciences, Linköping University
581 83 Linköping, Sweden
