# Supplementary material for: Effects of a waiting list control design on alcohol consumption among online help-seekers: protocol for a randomised controlled trial
Source: BMJ Open. 2021 Aug 26;11(8):e049810. doi: 10.1136/bmjopen-2021-049810 (PMC8395291; doi:10.1136/bmjopen-2021-049810)
Supplement: Supplementary data [file bmjopen-2021-049810supp002.pdf]

APPENDIX B - QUESTIONNAIRES

BASELINE AND FOLLOW-UP

1. (Baseline only) How old are you? (numeric)

2. (Baseline only) To assess risks from alcohol consumption, we need to know your biological sex?
   a. Female
   b. Male

3. In the past month, how often did you have six or more drinks on one occasion? (numeric)

4. Thinking about the past week, how many standard drinks did you have on: (numeric)
   a. Monday
   b. Tuesday
   c. Wednesday
   d. Thursday
   e. Friday
   f. Saturday
   g. Sunday

Note: A visual guide will be presented to participants with the definition of a standard drink.

The following questions are designed to identify how you personally feel about your drinking right now. Please think about your current situation and drinking habits, even if you have given up drinking completely. Read each question below carefully and then decide whether you agree or disagree with the statements.

Note: The options for each question below are Strongly disagree, Disagree, Unsure, Agree, Strongly agree. The letter in the parenthesis after the question indicates which stage of change the item belongs to and will not be shown to participants (PC = Precontemplation, C = Contemplation, A = Action).

5. It is a waste of time thinking about my drinking because I do not have a problem. (PC)
6. I enjoy my drinking but sometimes I drink too much. (C)
7. There is nothing seriously wrong with my drinking. (PC)
8. Sometimes I think I should quit or cut down my drinking. (C)
9. Anyone can talk about wanting to do something about their drinking, but I'm actually doing something about it. (A)
10. I am a fairly normal drinker. (PC)
11. My drinking is a problem sometimes. (C)
12. I am actually changing my drinking habits right now (either cutting down or quitting). (A)
13. I have started to carry out a plan to cut down or quit drinking. (A)
14. There is nothing I really need to change about my drinking. (PC)
15. Sometimes I wonder if my drinking is out of control. (C)
16. I am actively working on my drinking problem. (C)
