# Supplementary material for: Effects of a waiting list control design on alcohol consumption among online help-seekers: protocol for a randomised controlled trial
Source: BMJ Open. 2021 Aug 26;11(8):e049810. doi: 10.1136/bmjopen-2021-049810 (PMC8395291; doi:10.1136/bmjopen-2021-049810)
Supplement: Supplementary data [file bmjopen-2021-049810supp003.pdf]

APPENDIX C – DEBRIEFING EMAIL

The following email will be sent to the waiting list group after study completion.

Thank you for taking part in the "Digital support for alcohol" study.

The point of the study was to explore if being told to wait for the alcohol support tool made a difference to your alcohol consumption. Therefore, the material you received when you signed up for the study was the support tool which the other group also received. They were however told that they were given the support tool immediately.

This research is important because many studies use a design where participants are asked to wait before they get a new treatment, and it is uncertain what the effects of being told to wait are.

If you have questions about the study and its design, you are welcome to contact the primary investigator Marcus Bendtsen (see below for details).

If you want more support for change of your alcohol consumption you may find the information on this website helpful: Rethinking Drinking (https://www.rethinkingdrinking.niaaa.nih.gov/).

Contact details
Dr. Marcus Bendtsen, marcus.bendtsen@liu.se, 013-28 69 75
Docent in Medical Informatics
Senior Lecturer in Experimental Social Medicine and Public Health
Department of Health, Medicine and Caring Sciences, Linköping University
581 83 Linköping, Sweden
