# Supplementary material for: Effects of a waiting list control design on alcohol consumption among online help-seekers: protocol for a randomised controlled trial
Source: BMJ Open. 2021 Aug 26;11(8):e049810. doi: 10.1136/bmjopen-2021-049810 (PMC8395291; doi:10.1136/bmjopen-2021-049810)
Supplement: Author's manuscript [file bmjopen-2021-049810.draft_revisions.pdf]

BMJ Open

BMJ Open is committed to open peer review. As part of this commitment we make the peer review history of every article we publish publicly available.

When an article is published we post the peer reviewers' comments and the authors' responses online. We also post the versions of the paper that were used during peer review. These are the versions that the peer review comments apply to.

The versions of the paper that follow are the versions that were submitted during the peer review process. They are not the versions of record or the final published versions. They should not be cited or distributed as the published version of this manuscript.

BMJ Open is an open access journal and the full, final, typeset and author-corrected version of record of the manuscript is available on our site with no access controls, subscription charges or pay-per-view fees (http://bmjopen.bmj.com).

If you have any questions on BMJ Open's open peer review process please email info.bmjopen@bmj.com

BMJ Open

Effects of a waiting list control design on alcohol consumption among online help-seekers: protocol for a randomised controlled trial

Journal:	*BMJ Open*
Manuscript ID	bmjopen-2021-049810
Article Type:	Protocol
Date Submitted by the Author:	02-Feb-2021

Complete List of Authors:	Bendtsen, Marcus; Linköping University<br>Ulfsdotter Gunnarsson, Katarina; Linköping University<br>McCambridge, Jim; University of York, Department of Health Sciences

Keywords:	STATISTICS & RESEARCH METHODS, PUBLIC HEALTH, PREVENTIVE MEDICINE

SCHOLARONE™
Manuscripts

I, the Submitting Author has the right to grant and does grant on behalf of all authors of the Work (as defined in the below author licence), an exclusive licence and/or a non-exclusive licence for contributions from authors who are: i) UK Crown employees; ii) where BMJ has agreed a CC-BY licence shall apply, and/or iii) in accordance with the terms applicable for US Federal Government officers or employees acting as part of their official duties; on a worldwide, perpetual, irrevocable, royalty-free basis to BMJ Publishing Group Ltd ("BMJ") its licensees and where the relevant Journal is co-owned by BMJ to the co-owners of the Journal, to publish the Work in this journal and any other BMJ products and to exploit all rights, as set out in our licence.

The Submitting Author accepts and understands that any supply made under these terms is made by BMJ to the Submitting Author unless you are acting as an employee on behalf of your employer or a postgraduate student of an affiliated institution which is paying any applicable article publishing charge ("APC") for Open Access articles. Where the Submitting Author wishes to make the Work available on an Open Access basis (and intends to pay the relevant APC), the terms of reuse of such Open Access shall be governed by a Creative Commons licence – details of these licences and which Creative Commons licence will apply to this Work are set out in our licence referred to above.

Other than as permitted in any relevant BMJ Author's Self Archiving Policies, I confirm this Work has not been accepted for publication elsewhere, is not being considered for publication elsewhere and does not duplicate material already published. I confirm all authors consent to publication of this Work and authorise the granting of this licence.

For peer review only

EFFECTS OF A WAITING LIST CONTROL DESIGN ON ALCOHOL CONSUMPTION AMONG ONLINE HELP-SEEKERS: PROTOCOL FOR A RANDOMISED CONTROLLED TRIAL

Marcus Bendtsen[1,*], Katarina Ulfsdotter Gunnarson[1], Jim McCambridge[2]

[1] Department of Health, Medicine and Caring Sciences, Linköping University, Sweden.
[2] Department of Health Sciences, University of York.

*Corresponding author
Marcus Bendtsen (marcus.bendtsen@liu.se)
Department of Health, Medicine and Caring Sciences, Division of Society and Health
Linköping University, 581 83 Linköping, registrator@liu.se, +46 13 28 10 00

Word count: 3270

For peer review only

ABSTRACT

Introduction: Sparse attention has been given to the design of control conditions in trials, despite their important role as contrasts for novel treatments, and thus as a key determinant of effect sizes. This undermines valid inferences on effect estimates in trials, which are fundamentally comparative in nature. Such challenges to understanding also makes generalisation of effect estimates complex, for example it may not be clear to what degree real-world alternatives to the novel treatments in pragmatic trials are similar to the control conditions studied. The present study aims to estimate the effects of being allocated to a waiting list control condition.

Methods and analysis: Individuals searching online for help to reduce their drinking will be invited to take part in a study. Individuals aged 18 or older, who in the past month consumed 6 or more drinks on one occasion, or consumed 10 or more drinks the past week, will be eligible to participate. Both groups will receive identical feedback and advice on behaviour change, however, one group will be informed that they have to wait one month for the intervention materials. One month post-randomisation, participants will receive an email with the follow-up questionnaire measuring the primary outcomes: (1) frequency of heavy episodic drinking (defined as at study entry) in the past month; and (2) overall past week alcohol consumption. Differences between groups will be analysed using negative binomial regression models estimated using Bayesian inference. Recruitment will begin in April 2020. A Bayesian group sequential design will be employed to determine when to end enrolment (expected to be between 500 and 1500 individuals).

Ethics and dissemination: The study was approved by the Swedish Ethical Review Authority on 2021-01-25 (Dnr 2020-06267). Findings will be disseminated in open access peer-reviewed journals no later than 2023.

Registration: Prospectively registered on 2021-02-01 (ISRCTN14959594).

ARTICLE SUMMARY

STRENGTHS AND LIMITATIONS OF THIS STUDY

- Sparse attention has been given to the effects of different control conditions.
- Use of a double blind randomised controlled design allows for a valid estimate of the effects of being allocated to a waiting list control.
- The Bayesian group sequential design will allow for recruitment to not last longer than necessary, minimizing the number of participants exposed to the necessary deception involved in the study.
- The trial is an online trial, which usually suffer from high rates of attrition.

For peer review only

INTRODUCTION

Effects estimated in randomised controlled trials (RCTs) should always be understood as contrasts between allocated conditions [1]. Since effects are contrasts, estimates are affected by either side of the comparison. While much effort is usually placed in designing and describing the novel treatment side of the contrast, typically a fraction of this attention is given to the control side. There is sparse attention to the design of control conditions in trials in the research literature [2–6]. Not only does this potentially introduce bias, and certainly complicates the interpretation of the effects estimated, it also makes generalisability problematic, with implications for research use in decision-making.

To illustrate the complex nature of the generalisability issues, we borrow notation from the potential outcomes framework [7]. Let Y_x represent the outcome for an individual if the individual would hypothetically receive treatment x. There may be a range of different treatments available, and depending on which treatment an individual actually receives, different outcomes will follow. In a 2-arm RCT, the two *potential* outcomes for participants are Y_0 and Y_1 , i.e., the outcomes realised if they are allocated to the control condition ($x = 0$) or the intervention condition ($x = 1$). The individual level causal effect of the intervention is defined as $Y_1 - Y_0$, that is, the potential outcome under the intervention condition minus the potential outcome under the control condition (or some other mode of contrast, e.g. risk or odds). Of course, when using between-subjects designs it will be impossible to know both Y_1 and Y_0 for the same individual, thus RCTs are generally focused on the population level causal effect. With continuous outcomes, this is commonly the expected difference $E[Y_1] - E[Y_0]$ between the two conditions. We rely on the license given to us by virtue of randomisation to claim that these are unconfounded estimates with sufficiently large numbers, protecting the internal validity of our estimates.

Ideally, $E[Y_1] - E[Y_0]$ would be an estimate which arises from an effectiveness or pragmatic trial rather than an efficacy or explanatory trial [8,9], which can be interpreted as the effect anticipated in a real-world roll-out. Y_1 could be a novel potential outcome which we may offer to individuals, and it may be close to what was estimated in the trial if the study inclusion criteria were similar to routine practice, there were no marked bias, and if treatment protocols are followed. However, a problem arises if Y_0 is not the potential outcome which would be the alternative to Y_1 in the real world. For instance, if Y_0 represents the potential outcome of being placed on a waiting list [10], then that is not an outcome which is available to individuals in a real-world setting, except in those situations where there are waiting lists for treatment. To avoid any such problem the control condition in the trial needs to be identical to usual care. If being placed on a waiting list in a study affects individuals in any way differently than do the real-world alternatives to Y_1, then the external validity of the contrast $E[Y_1] - E[Y_0]$ should be questioned, and measures need to be taken to facilitate the interpretation of the observed effect.

WAITING LIST CONTROLS

Rather than withholding treatment from controls in RCTs altogether, it has been argued that it is more ethical to offer the treatment to controls after the trial period [10] – i.e. placing participants on a waiting list. This type of control condition is commonly used in behavioural and rehabilitation treatments [11], altering the nature of Y_0. It is not well understood how individuals react to these decisions. For example, in one smoking cessation trial some control participants had negative feelings about having to wait, and decided to quit later [12]. Researchers have long been concerned with feelings of disappointment about allocation to control conditions in trials more broadly, but we know little about how study participants subsequently act on them [13,14]. It remains unclear if these reactions translate into any markedly biased effect estimates.

Indirect evidence suggests that in many circumstances we may expect participants in control conditions to change more than they ordinarily would if not involved in a study. It has for instance been found that alcohol

For peer review only - http://bmjopen.bmj.com/site/about/guidelines.xhtml

consumption tends to decrease in control groups by approximately 20% in alcohol intervention trials [15–17]. This change is likely due to a combination of several factors, including regression to the mean [18], assessment effects [19], and other forms of research participation effects [20–22]. A network meta-analysis of cognitive behaviour therapy for depression [23], found in contrast that participants in waiting list control groups improved less than expected. This is line with thinking that when patients are placed on waiting lists, they are to some extent compliant with the implicit direction; they wait, and avoid instigating changes [24].

A direct estimation of the effects of a waiting list control design on alcohol consumption was conducted among 185 hazardous or harmful drinkers recruited through newspaper advertisements in Canada [25]. A 2-arm parallel groups RCT was employed, in which both groups received the same brief alcohol intervention, but one group were told that they had been allocated to the intervention arm, and the other told they were allocated to the a waiting list arm. This exploratory trial found an interaction between being allocated to the waiting list group and the action subscale of the readiness to change questionnaire [26,27]. This suggested that those in the waiting list group who rated themselves above the median on the action subscale (thus being more ready for action) reported consuming more alcohol in comparison to their counterparts in the intervention group (approximately 6 drinks more per week and 1.3 more drinks on the occasion when they drank the most). They thus waited to reduce their drinking. While findings from the trial were mixed, it generated hypotheses important to study further. The current trial described in this protocol will address these hypotheses with a larger sample size in an online recruitment setting.

OBJECTIVES

Using a 2-arm (1:1) randomised controlled trial, the objective of this study is to estimate the effects of being allocated to a waiting list control group on alcohol consumption. The primary effects being estimated are the total effect and moderation by readiness to change.

METHODS AND ANALYSIS

This protocol contains relevant items from the Standard Protocol Items: Recommendations for Interventional Trials (SPIRIT) [28]. The trial has been prospectively registered in the ISRCTN registry on 2021-02-01 (ISRCTN14959594), and has received ethical approval by the Swedish Ethical Review Authority on 2021-01-25 (Dnr 2020-06267).

STUDY SETTING, ELIGIBILITY CRITERIA AND RECRUITMENT

Participants will be recruited through online advertisements (Google Ads), using language targeting to include individuals using Google's services in English (including search and gmail). Keywords will be selected to target those seeking help to drink less, e.g. "How do I drink less", "I drink too much", and "Support for drinkers". The adverts will be designed to target individuals who are willing to take part in a trial of a self-help alcohol intervention. An example advert is shown in Figure 1, and study information presented to individuals who click on the advert can be found in Appendix A. Recruitment will begin in April 2020 and last for no more than 24 months (see Sample Size).

INSERT FIGURE1 HERE

Figure 1 - Example advert shown to individuals who search online for help to drink less

Individuals who consent to take part in the trial will be asked to complete a baseline questionnaire (see Appendix B). Individuals will first be asked to state their age and sex, the number of times during the past month they consumed 6 or more drinks on one occasion, and the number of drinks consumed in the past week. Individuals will be included if they report having consumed 6 or more drinks on one occasion at least

For peer review only - http://bmjopen.bmj.com/site/about/guidelines.xhtml

once in the past month or having consumed more than 9 drinks in the past week. Individuals younger than 18 years old will be excluded. Those not excluded will be asked to complete the readiness to change questionnaire. After completing the questionnaire, individuals will be asked to leave an email address to which further information about study participation will be sent. The email will be sent immediately and will include a brief text explaining that a link should be clicked in the email to continue participation in the study. Individuals who click on the link in the email will be randomised to either the intervention group (IG) or the waiting list control group (WL).

INTERVENTIONS

Participants in both groups (IG and WL) will receive the same personalised feedback and advice based on the baseline questionnaire. The material, which has been developed from our previous research [19,29–35] and is based around social cognitive theories of health behaviour [36], includes: (1) feedback on current consumption in relation to recommendations, (2) content to increase motivation and self-efficacy, and (3) advice on what to do to reduce drinking and avoid environmental triggers.

Preceding the feedback and advice will be a page with study information which will be different between groups; this is the experimental contrast in the present study.

Text to IG group
There are two groups in this study. You are in the group which receives immediate feedback and support tailored to the responses to the questionnaire you previously completed. This material has been designed for people who are concerned about their drinking. [Button: Take me to the feedback].

Text to WL group
There are two groups in this study. You are in the group which will have to wait for the feedback and support material. We will contact you again in four weeks, and at that time you will be given the material. In the meantime, we have generated a report based on your responses to the questionnaire previously completed. [Button: Take me to the feedback].

OUTCOMES

Primary:

1. Frequency of heavy episodic drinking in the past month
2. Overall consumption the past week

Secondary:

3. Readiness to change

The two primary outcomes have been selected to be similar to the outcomes used in the exploratory trial upon which this trial has been modelled [25], while at the same time being part of the proposed core outcome set for brief alcohol interventions [37,38]. Frequency of heavy episodic drinking will be defined as the number of times in the past month the participant has consumed 6 or more drinks on one occasion. Number of drinks consumed in the past week will be assessed by asking participants to report their consumption day-by-day.

The secondary outcome, readiness to change, will be measured using the readiness to change questionnaire (treatment version) [26,27]. While our primary use of this scale is for assessing moderation effects (using the baseline assessment), we are also interested in estimating effects of the WL condition on readiness to change directly.

For peer review only

FOLLOW-UP

One month post randomisation, participants will be sent an email including a link to the follow-up questionnaire (see Appendix B). Participants will be sent two reminders three days apart. If no response has been recorded three days after the final reminder, a fourth email will be sent with the frequency of heavy episodic drinking in the past month measure embedded in the email. Reminders will only be sent to participants who have not yet responded to the two primary alcohol consumption outcomes. Once data collection is complete, an email will be sent to the WL group with information about the deception involved in the study (Appendix C). A participant timeline is shown in Figure 2.

INSERT FIGURE2 HERE

Figure 2 - Participant timeline (SPIRIT figure)

ALLOCATION AND BLINDING

Block randomisation will be used with randomly permuted block sizes of 2 and 4 to ensure equal size groups. Randomisation will be automatic and computerised on the backend server, thus neither research personnel nor participants will be able to influence allocation. Participants will be blind to the true nature of the study. Research personnel will be blind to allocation.

STATISTICAL METHODS

All analyses will be conducted according to intention-to-treat principles, with all participants analysed in the groups to which they were randomised. Analyses will be done using available data with missing data imputed. Attrition analyses will explore the plausibility of the implicit missing at random (MAR) assumption underlying the analyses. Model parameters will be interpreted by inspecting marginal posterior distributions using Bayesian inference (see Sample Size for priors) [39], and imputation will be done within the Markov Chain Monte Carlo iterations during inference. We will use R version 3.6 with the RStan library for all analyses [40].

PRIMARY ANALYSES

The analysis of primary and secondary outcomes will be conducted through regression models in which each outcome will be regressed against group allocation. One regression model per outcome will be created. Negative binomial regression will be used for both primary outcomes and linear regression will be used for readiness to change scores (precontemplation, contemplation and action). A multinomial regression model will also be estimated for stage of change designation (i.e. highest arithmetical score among the three sub scales with ties preferring the stage farther along the continuum of change [26,27]). All regression models will be adjusted for age, sex and each respective outcome measured at baseline.

EXPLORATORY ANALYSES

Effect modification analyses of group allocation and readiness to change scores (precontemplation, contemplation and action) on the primary outcomes will be analysed by adding interaction terms to the respective regression models. We will estimate separate models for each stage interaction, and a combined model with all stages interacting with allocation using shrinkage priors [41]. We will also explore effect modification with respect to group allocation and stage of change designation on the primary outcomes.

Subgroup analyses will be conducted among those who were screened at-risk with respect to the two primary outcomes, i.e. heavy episodic drinking among those reporting having consumed 6 or more drinks on one or

For peer review only - http://bmjopen.bmj.com/site/about/guidelines.xhtml

more occasions over the past month, and overall weekly consumption among those who reported having consumed more than 9 drinks the past week at baseline.

MEDIATOR ANALYSES

We will estimate the degree to which the effect of allocation on the primary outcomes is mediated through two variables: (1) accessing the intervention materials and (2) time spent on the intervention materials. We will use a causal inference framework [42–44], using Bayesian inference to estimate the natural direct effect and natural indirect effect (as per the definitions of Pearl [44]). We will report on the posterior distributions of these two estimates, as well as the proportion of the total effect which is accounted for by the natural indirect effect. We will also explore if readiness to change moderates any of these effects.

ATTRITION ANALYSES

The available data and imputation approach used in the primary analyses may result in biased intention-to-treat estimates if data is not MAR. We will therefore seek evidence against the MAR assumption in two attrition analyses. First, since late responders to follow-up may be more alike non-responders than early responders are, we will investigate if outcomes are systematically different given the number of reminders needed to collect follow-up data. Second, we will explore if responders and non-responders are systematically different with respect to baseline characteristics, using logistic regression models with shrinkage priors.

SAMPLE SIZE

The trial will use a Bayesian group sequential design to monitor recruitment with interim analyses planned for every 25 participants completing the follow-up questionnaire [45–47]. The primary outcomes will be modelled following the analysis plan presented earlier, and each covariate representing group allocation will be assessed for evidence of effect or futility. Covariates representing the interaction between the stages of readiness for change and group allocation will also be monitored (using separate models for each stage interaction). Let β_i represent the covariates of interest, and D the data available, then for each β_i the target criteria will be:

- **Effect**: $p(\beta_i > 0|D) > 97.5\%$ or $p(\beta_i < 0|D) > 97.5\%$ (i.e. the effect is greater (or less) than 0 with a probability greater than 97.5%).

- **Futility**: $p(\log(\frac{1}{1.10}) < \beta_i < \log(1.10)|D) > 95.0\%$ (i.e. the incidence rate ratio is greater than 1/1.10 and less than 1.10 with a probability greater than 95%).

For the effect criterion, we will use a sceptical normal prior for covariates (mean = 0 and standard deviation = 1.0), and a wider prior will be used for the futility criterion (mean = 0 and standard deviation = 2.0).

The criteria above should be viewed as targets, thus at each interim analysis we will evaluate each criterion for each covariate and make a decision if we believe that recruitment should end. Simulations indicate that we will require a sample size in the range of 500 to 1500 participants. Recruitment will not exceed 24 months.

PATIENT AND PUBLIC INVOLVEMENT STATEMENT

Patients were not involved in the planning of this study.

For peer review only

DISCUSSION

Trials are usually advertised as important for research, but they may also be seen as an opportunity for prospective participants to get access to novel treatments. Trials do not primarily advertise the conditions which lead to Y_0 , i.e., recruitment material never says “Do you want to be on a waiting list?”. Participants have likely found the trial, and enrolled, motivated by the ways in which the trial may benefit them. This may be especially the case in online studies when participants actively engage with recruitment procedures, rather than simply respond to in-person contacts from researchers that they have not initiated. On the other hand, the effects of being informed that one has to wait, as investigated here, may be less pronounced in online studies [48–52]. Regardless of the setting, the unintended consequences of telling participants that they will have to wait is the motivation for the present study, which is designed to uncover some of these consequences in the chosen setting .

LIMITATIONS

Online studies of alcohol interventions commonly suffer from high levels of attrition, particularly at follow-up, and we expect the primary limitation of this study to be high attrition rates. To reduce attrition, we will only randomise participants after they have confirmed their email address, which helps to ensure that we have a valid email address for each randomised participant. We will use reminders over a short time span to keep participants aware that it is important that they respond, and finally make it easy for them to respond by including one of the primary outcome measures in the body of the email. The Bayesian group sequential design will ensure that we collect data for as long as is necessary to fulfil the criteria (see Sample Size), which will ensure that we have enough samples for our analyses. However, this will not protect against differential attrition, and thus does not protect against attrition bias, so we will need to study this risk carefully.

ETHICS AND DISSEMINATION

The main ethical concern in this trial is the use of deception - required to not reveal to prospective participants that it is the effects of allocation which are being studied. Routine use of deception in research is not recommended, however, considering the widespread use of waiting list designs, understanding their effects was considered important and justified the risks induced by deception. The study was approved by the Swedish Ethical Review Authority on 2021-01-25 (Dnr 2020-06267). Data collection will begin in April 2021 and last no longer than 24 months. The dataset will be made available to researchers upon reasonable request. Findings will be published in peer-reviewed journals and presented at relevant conferences no later than 2023.

REFERENCES

1. Barkauskas VH, Lusk SL, Eakin BL. Selecting Control Interventions for Clinical Outcome Studies. West J Nurs Res. 2005 Apr;27(3):346–63.

2. Cook TD, Campbell DT. Quasi-Experimentation: Design and Analysis Issues for Field Settings. Houghton Mifflin; 1979.

3. Rosnow RL, Rosenthal R. People studying people: Artifacts and ethics in behavioral research. New York: W.H. Freeman; 1997.

4. Freedland KE, King AC, Ambrosius WT, Mayo-Wilson E, Mohr DC, Czajkowski SM, et al. The selection of comparators for randomized controlled trials of health-related behavioral interventions: recommendations of an NIH expert panel. J Clin Epidemiol. 2019 Jun;110:74–81.

5. Freedland KE. Demanding Attention: reconsidering the role of attention control groups in behavioral intervention research. Psychosom Med. 2013;75(2):100–2.

For peer review only - http://bmjopen.bmj.com/site/about/guidelines.xhtml

6. Freedland KE, Mohr DC, Davidson KW, Schwartz JE. Usual and Unusual Care: existing practice control groups in randomized controlled trials of behavioral interventions. Psychosom Med. 2011 May;73(4):323–35.

7. Rubin DB. Estimating causal effects of treatments in randomized and nonrandomized studies. J Educ Psychol. 1974;66(5):688–701.

8. Singal AG, Higgins PDR, Waljee AK. A Primer on Effectiveness and Efficacy Trials. Clin Transl Gastroenterol. 2014 Jan;5(1):e45.

9. Roland M, Torgerson DJ. Understanding controlled trials: What are pragmatic trials? BMJ. 1998 Jan 24;316(7127):285–285.

10. Elliott S., Brown JS. What are we doing to waiting list controls? Behav Res Ther. 2002 Sep;40(9):1047–52.

11. Hart T, Bagiella E. Design and Implementation of Clinical Trials in Rehabilitation Research. Arch Phys Med Rehabil. 2012 Aug;93(8):S117–26.

12. Müssener U, Linderoth C, Bendtsen M. Exploring the Experiences of Individuals Allocated to a Control Setting: Findings From a Mobile Health Smoking Cessation Trial. JMIR Hum Factors. 2019;6(2):e12139.

13. McCambridge J, Sorhaindo A, Quirk A, Nanchahal K. Patient preferences and performance bias in a weight loss trial with a usual care arm. Patient Educ Couns. 2014 May;95(2):243–7.

14. Howick J. Questioning the Methodologic Superiority of 'Placebo' Over 'Active' Controlled Trials. Am J Bioeth. 2009 Sep 11;9(9):34–48.

15. Jenkins RJ, McAlaney J, McCambridge J. Change over time in alcohol consumption in control groups in brief intervention studies: systematic review and meta-regression study. Drug Alcohol Depend. 2009 Feb;100(1–2):107–14.

16. Jenkins RJ, McAlaney J, McCambridge J. Corrigendum to "Change over time in alcohol consumption in control groups in brief intervention studies: Systematic review and meta-regression study" [Drug Alcohol Depend. 100 (2009) 107–114]. Drug Alcohol Depend. 2010 Apr;108(1–2):151.

17. Moyer A, Finney JW. Outcomes for untreated individuals involved in randomized trials of alcohol treatment. J Subst Abuse Treat. 2002 Oct;23(3):247–52.

18. Linden A. Assessing regression to the mean effects in health care initiatives. BMC Med Res Methodol. 2013 Dec 28;13(1):119.

19. McCambridge J, Bendtsen M, Karlsson N, White IR, Nilsen P, Bendtsen P. Alcohol assessment and feedback by email for university students: Main findings from a randomised controlled trial. Br J Psychiatry. 2013;203(5):334–40.

20. McCambridge J, Witton J, Elbourne DR. Systematic review of the Hawthorne effect: New concepts are needed to study research participation effects. J Clin Epidemiol. 2014 Mar;67(3):267–77.

21. McCambridge J, Kypri K, Elbourne D. Research participation effects: a skeleton in the methodological cupboard. J Clin Epidemiol. 2014 Aug;67(8):845–9.

22. McCambridge J, Kypri K, McElduff P. Regression to the mean and alcohol consumption: A cohort study exploring implications for the interpretation of change in control groups in brief intervention trials. Drug Alcohol Depend. 2014 Feb;135:156–9.

23. Furukawa TA, Noma H, Caldwell DM, Honyashiki M, Shinohara K, Imai H, et al. Waiting list may be a nocebo condition in psychotherapy trials: a contribution from network meta-analysis. Acta Psychiatr Scand. 2014 Sep;130(3):181–92.

For peer review only

24. Miller W, Rollnick S. Motivational Interviewing: Preparing People for Change. New York: Guilford Press; 2002.

25. Cunningham JA, Kypri K, McCambridge J. Exploratory randomized controlled trial evaluating the impact of a waiting list control design. BMC Med Res Methodol. 2013 Dec;13(1):150.

26. Rollnick S, Heather N, Gold R, Hall W. Development of a short “readiness to change” questionnaire for use in brief, opportunistic interventions among excessive drinkers. Addiction. 1992 May;87(5):743–54.

27. Heather N, Hönekopp J. A revised edition of the Readiness to Change Questionnaire [Treatment Version]. Addict Res Theory. 2008 Jan 11;16(5):421–33.

28. Chan A-W, Tetzlaff JM, Altman DG, Laupacis A, Gøtzsche PC, Krleža-Jerić K, et al. SPIRIT 2013 Statement: Defining Standard Protocol Items for Clinical Trials. Ann Intern Med. 2013 Feb 5;158(3):200.

29. Bendtsen P, McCambridge J, Bendtsen M, Karlsson N, Nilsen P. Effectiveness of a Proactive Mail-Based Alcohol Internet Intervention for University Students: Dismantling the Assessment and Feedback Components in a Randomized Controlled Trial. J Med Internet Res. 2012 Oct 31;14(5):e142.

30. McCambridge J, Bendtsen P, Bendtsen M, Nilsen P. Alcohol email assessment and feedback study dismantling effectiveness for university students (AMADEUS-1): study protocol for a randomized controlled trial. Trials. 2012 Dec 6;13(1):49.

31. McCambridge J, Bendtsen M, Karlsson N, White IR, Bendtsen P. Alcohol assessment & feedback by e-mail for university student hazardous and harmful drinkers: Study protocol for the AMADEUS-2 randomised controlled trial. BMC Public Health. 2013;13(1):949.

32. Bendtsen P, Bendtsen M, Karlsson N, White IR, McCambridge J. Online Alcohol Assessment and Feedback for Hazardous and Harmful Drinkers: Findings From the AMADEUS-2 Randomized Controlled Trial of Routine Practice in Swedish Universities. J Med Internet Res. 2015;17(7):e170.

33. Bendtsen M. An Electronic Screening and Brief Intervention for Hazardous and Harmful Drinking Among Swedish University Students: Reanalysis of Findings From a Randomized Controlled Trial Using a Bayesian Framework. J Med Internet Res. 2019;21(12):e14420.

34. Bendtsen M. Electronic Screening for Alcohol Use and Brief Intervention by Email for University Students: Reanalysis of Findings From a Randomized Controlled Trial Using a Bayesian Framework. J Med Internet Res. 2019;21(11):e14419.

35. Bendtsen M, McCambridge J. Reducing Alcohol Consumption Among Risky Drinkers in the General Population of Sweden Using an Interactive Mobile Health Intervention: Protocol for a Randomized Controlled Trial. JMIR Res Protoc. 2019;8(4):e13119.

36. Conner M, Norman P. Predicting Health Behavior: Research and Practice with Social Cognition Models. 2005.

37. Shorter GW, Bray JW, Giles EL, O’Donnell AJ, Berman AH, Holloway A, et al. The Variability of Outcomes Used in Efficacy and Effectiveness Trials of Alcohol Brief Interventions: A Systematic Review. J Stud Alcohol Drugs. 2019 May;80(3):286–98.

38. Shorter GW, Heather N, Bray JW, Berman AH, Giles EL, O’Donnell AJ, et al. Prioritization of Outcomes in Efficacy and Effectiveness of Alcohol Brief Intervention Trials: International Multi-Stakeholder e-Delphi Consensus Study to Inform a Core Outcome Set. J Stud Alcohol Drugs. 2019 May;80(3):299–309.

39. Bendtsen M. A Gentle Introduction to the Comparison Between Null Hypothesis Testing and Bayesian Analysis: Reanalysis of Two Randomized Controlled Trials. J Med Internet Res. 2018;20(10):e10873.

40. Team SD. RStan: the R interface to Stan. 2020.

For peer review only

41. Piironen J, Vehtari A. Sparsity information and regularization in the horseshoe and other shrinkage priors. Electron J Stat. 2017;11(2):5018–51.

42. Imai K, Keele L, Tingley D. A General Approach to Causal Mediation Analysis. Psychol Methods. 2010;15(4):309–34.

43. Pearl J. Causality. Causality: Models, Reasoning, and Inference, Second Edition. Cambridge: Cambridge University Press; 2009.

44. Pearl J. Interpretation and identification of causal mediation. Psychol Methods. 2014;19(4):459–81.

45. Gsponer T, Gerber F, Bornkamp B, Ohlssen D, Vandemeulebroecke M, Schmidli H. A practical guide to Bayesian group sequential designs. Pharm Stat. 2014;13(1):71–80.

46. Berry DA. Bayesian clinical trials. Nat Rev Drug Discov. 2006 Jan;5(1):27–36.

47. Bendtsen M. The P Value Line Dance: When Does the Music Stop? J Med Internet Res. 2020;22(8):e21345.

48. McCambridge J, Wilson A, Attia J, Weaver N, Kypri K. Randomized trial seeking to induce the Hawthorne effect found no evidence for any effect on self-reported alcohol consumption online. J Clin Epidemiol. 2019 Apr;108:102–9.

49. Kypri K, Wilson A, Attia J, Sheeran P, Miller P, McCambridge J. Social Desirability Bias in the Reporting of Alcohol Consumption: A Randomized Trial. J Stud Alcohol Drugs. 2016 May;77(3):526–31.

50. McCambridge J. From question-behaviour effects in trials to the social psychology of research participation. Psychol Health. 2015 Jan 2;30(1):72–84.

51. Kypri K, Wilson A, Attia J, Sheeran PJ, McCambridge J. Effects of study design and allocation on self-reported alcohol consumption: randomized trial. Trials. 2015 Dec;16(1):127.

52. Kypri K, Bowe SJ, Karlsson N, McCambridge J. Enrolment-latency in randomized behavior change trials: individual participant data meta-analysis showed association with attrition but not effect-size. J Clin Epidemiol. 2020 Feb;118:55–9.

For peer review only

AUTHORS' CONTRIBUTIONS

MB and JM defined the study objectives and designed the trial. All authors were involved in developing the intervention materials. MB and KUG are responsible for data collection and statistical analysis. All authors are involved in interpreting findings and all authors read and approved the protocol manuscript.

FUNDING

This research received no specific grant from any funding agency in the public, commercial or not-for-profit sectors.

COMPETING INTERESTS

MB owns a private company (Alexit AB) which disseminates digital lifestyle interventions such as the one offered to participants in this trial. Alexit AB had no part in funding, planning, or execution of this study.

DATA AVAILABILITY STATEMENT

The dataset will be made available to researchers upon reasonable request.

FIGURE LEGENDS

Figure 3 - Example advert shown to individuals who search online for help to drink less

Figure 2 - Participant timeline (SPIRIT figure)

For peer review only

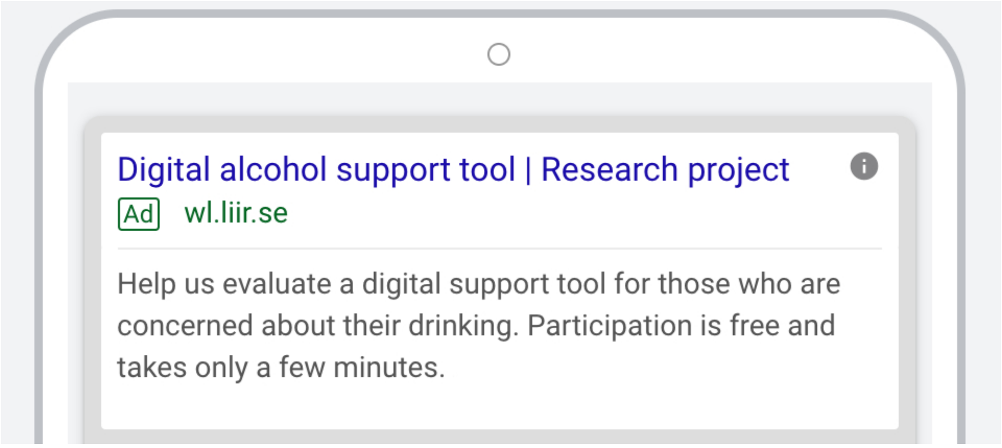

Example advert shown to individuals who search online for help to drink less

	**STUDY PERIOD**			
	**Enrolment**	**Allocation**	**Post-allocation**	**Close-out**
**TIMEPOINT**	**0**	**0**	***Access to feedback and support material***	***1-month***
**ENROLMENT:**				
Informed consent	X			
Eligibility screen	X			
Allocation		X		
Debriefing				X
**INTERVENTIONS:**				
Intervention group (IG)			↔	
Waiting list group (WL)			↔	
**ASSESSMENTS:**				
Baseline questionnaire	X			
Follow-up questionnaire				X
Accessing material			X	X
Time spent on material			X	

For peer review only - http://bmjopen.bmj.com/site/about/guidelines.xhtml

APPENDIX A – INFORMED CONSENT MATERIALS

When clicking on the online advert, participants will be shown the following information about the study.

Do you want to participate in the "Digital support for alcohol" study?

Thank you for showing interest in this study. By pressing the button "I consent to take part in this study" you are consenting to take part in the study described below.

Why are we doing this study and why do you want me to participate? We have developed a digital support tool which is tailored to individual responses to a questionnaire. The tool has been designed for people who are concerned about their drinking. In order to evaluate if the tool is effective, we are asking people who search online to be part of this study.

This research project is conducted at Linköping university in Sweden, please find contact details for the primary investigator below.

What is expected of me? If you take part in the study you will first be asked to respond to a 5 minute questionnaire. Thereafter you will be asked to leave an email address to which we can send more information about the study. You will be randomly assigned to either get direct access to the tool or wait one month before being given access. One month after having signed up for the study we will send an email to you with a link to a short follow-up questionnaire.

Will I be taking any risks by participating in this study? Participating in this study will give you access to a novel digital support tool which has been designed to help people concerned about their drinking. However, you may find that the support given to you is inadequate and does not help you, and you may be de-motivated by this. You should be aware of this before deciding to take part in this study. We are however not asking you to refrain from any other support which may be available to you. For instance, you may find the information available at this website helpful: Rethinking Drinking (https://www.rethinkingdrinking.niaaa.nih.gov/).

Will I be leaving any personal information? We will collect data from you about your age, biological sex, alcohol consumption, and your readiness to change. We will also collect an email address from you so that we can contact you for follow-up one month after enrolling. The data collection is for scientific research, and is therefore motivated by public interest (GDPR EU 2016/679, Prop. 2017/18:298). Your data will be treated with confidentiality and we will take measures to ensure that no unauthorized access is granted.

Linköping University will be responsible for your personal data. According to EU law, you retain the right to, without cost, be given access to your personal data and request that changes be made. You can also request that your personal data be deleted. Your rights for deletion of data is however restricted if the data is deemed necessary for the study to be conducted. If you want access to your personal data you should contact the primary investigator Marcus Bendtsen (see information below). The data protection officer can be contacted at dataskyddsombud@liu.se. If you are not satisfied with how your personal data has been handled, you can make a formal complain to the Swedish Authority for Privacy Protection.

Where will data be stored and who will have access? During the study period we will store all data on a database at Linköping university in Sweden. After the study period the data will be archived securely at Linköping university and removed from the database. Access to the data will at first be restricted to the research team, but will later be made available for other researchers by request. Please note that once the study period is complete, all email addresses will be deleted and data will therefore be anonymous.

Who is responsible for the study? Linköping university is responsible for the data collected in this study. If you have any questions about your data you can contact Dr. Marcus Bendtsen, (marcus.bendtsen@liu.se), or the data protection officer (dataskyddsombudet@liu.se). If you are not satisfied with the way your personal data has been handled, you can file a complaint at the Swedish Data Protection Authority.

How can I get more information about the results from this study? All results will be published in peer-reviewed journals and conferences no later than 2022.

Participation is voluntary. You can at any point decide to not be part of the study and you will not have to explain why.

For peer review only

Contact details

Dr. Marcus Bendtsen, marcus.bendtsen@liu.se, 013-28 69 75
Docent in Medical Informatics
Senior Lecturer in Experimental Social Medicine and Public Health
Department of Health, Medicine and Caring Sciences, Linköping University
581 83 Linköping, Sweden

For peer review only

APPENDIX B - QUESTIONNAIRES

BASELINE AND FOLLOW-UP

1. (Baseline only) How old are you? (numeric)

2. (Baseline only) To assess risks from alcohol consumption, we need to know your biological sex?
   a. Female
   b. Male

3. In the past month, how often did you have six or more drinks on one occasion? (numeric)

4. Thinking about the past week, how many standard drinks did you have on: (numeric)
   a. Monday
   b. Tuesday
   c. Wednesday
   d. Thursday
   e. Friday
   f. Saturday
   g. Sunday

Note: A visual guide will be presented to participants with the definition of a standard drink.

The following questions are designed to identify how you personally feel about your drinking right now. Please think about your current situation and drinking habits, even if you have given up drinking completely. Read each question below carefully and then decide whether you agree or disagree with the statements.

Note: The options for each question below are Strongly disagree, Disagree, Unsure, Agree, Strongly agree. The letter in the parenthesis after the question indicates which stage of change the item belongs to and will not be shown to participants (PC = Precontemplation, C = Contemplation, A = Action).

5. It is a waste of time thinking about my drinking because I do not have a problem. (PC)
6. I enjoy my drinking but sometimes I drink too much. (C)
7. There is nothing seriously wrong with my drinking. (PC)
8. Sometimes I think I should quit or cut down my drinking. (C)
9. Anyone can talk about wanting to do something about their drinking, but I'm actually doing something about it. (A)
10. I am a fairly normal drinker. (PC)
11. My drinking is a problem sometimes. (C)
12. I am actually changing my drinking habits right now (either cutting down or quitting). (A)
13. I have started to carry out a plan to cut down or quit drinking. (A)
14. There is nothing I really need to change about my drinking. (PC)
15. Sometimes I wonder if my drinking is out of control. (C)
16. I am actively working on my drinking problem. (C)

For peer review only

APPENDIX C – DEBRIEFING EMAIL

The following email will be sent to the waiting list group after study completion.

Thank you for taking part in the "Digital support for alcohol" study.

The point of the study was to explore if being told to wait for the alcohol support tool made a difference to your alcohol consumption. Therefore, the material you received when you signed up for the study was the support tool which the other group also received. They were however told that they were given the support tool immediately.

This research is important because many studies use a design where participants are asked to wait before they get a new treatment, and it is uncertain what the effects of being told to wait are.

If you have questions about the study and its design, you are welcome to contact the primary investigator Marcus Bendtsen (see below for details).

If you want more support for change of your alcohol consumption you may find the information on this website helpful: Rethinking Drinking (https://www.rethinkingdrinking.niaaa.nih.gov/).

Contact details

Dr. Marcus Bendtsen, marcus.bendtsen@liu.se, 013-28 69 75
Docent in Medical Informatics
Senior Lecturer in Experimental Social Medicine and Public Health
Department of Health, Medicine and Caring Sciences, Linköping University
581 83 Linköping, Sweden

For peer review only

SPIRIT

STANDARD PROTOCOL ITEMS: RECOMMENDATIONS FOR INTERVENTIONAL TRIALS

SPIRIT 2013 Checklist: Recommended items to address in a clinical trial protocol and related documents*

**Section/item**	**Item No**	**Description**	**Page Number on which item is reported**
**Administrative information**			
Title	1	Descriptive title identifying the study design, population, interventions, and, if applicable, trial acronym	1
Trial registration	2a	Trial identifier and registry name. If not yet registered, name of intended registry	2
	2b	All items from the World Health Organization Trial Registration Data Set	2
Protocol version	3	Date and version identifier	NA
Funding	4	Sources and types of financial, material, and other support	2,12
Roles and responsibilitie s	5a	Names, affiliations, and roles of protocol contributors	1
	5b	Name and contact information for the trial sponsor	NA
	5c	Role of study sponsor and funders, if any, in study design; collection, management, analysis, and interpretation of data; writing of the report; and the decision to submit the report for publication, including whether they will have ultimate authority over any of these activities	NA
	5d	Composition, roles, and responsibilities of the coordinating centre, steering committee, endpoint adjudication committee, data management team, and other individuals or groups overseeing the trial, if applicable (see Item 21a for data monitoring committee)	NA
**Introduction**			

For peer review only - http://bmjopen.bmj.com/site/about/guidelines.xhtml

Background and rationale	6a	Description of research question and justification for undertaking the trial, including summary of relevant studies (published and unpublished) examining benefits and harms for each intervention	3-4
	6b	Explanation for choice of comparators	3-4
Objectives	7	Specific objectives or hypotheses	4
Trial design	8	Description of trial design including type of trial (eg, parallel group, crossover, factorial, single group), allocation ratio, and framework (eg, superiority, equivalence, noninferiority, exploratory)	4
**Methods: Participants, interventions, and outcomes**			
Study setting	9	Description of study settings (eg, community clinic, academic hospital) and list of countries where data will be collected. Reference to where list of study sites can be obtained	4-5
Eligibility criteria	10	Inclusion and exclusion criteria for participants. If applicable, eligibility criteria for study centres and individuals who will perform the interventions (eg, surgeons, psychotherapists)	4-5
Interventions	11a	Interventions for each group with sufficient detail to allow replication, including how and when they will be administered	5
	11b	Criteria for discontinuing or modifying allocated interventions for a given trial participant (eg, drug dose change in response to harms, participant request, or improving/worsening disease)	NA
	11c	Strategies to improve adherence to intervention protocols, and any procedures for monitoring adherence (eg, drug tablet return, laboratory tests)	4-5
	11d	Relevant concomitant care and interventions that are permitted or prohibited during the trial	NA
Outcomes	12	Primary, secondary, and other outcomes, including the specific measurement variable (eg, systolic blood pressure), analysis metric (eg, change from baseline, final value, time to event), method of aggregation (eg, median, proportion), and time point for each outcome. Explanation of the clinical relevance of chosen efficacy and harm outcomes is strongly recommended	6

Participant timeline	13	Time schedule of enrolment, interventions (including any run-ins and washouts), assessments, and visits for participants. A schematic diagram is highly recommended (see Figure)	Figure 2
Sample size	14	Estimated number of participants needed to achieve study objectives and how it was determined, including clinical and statistical assumptions supporting any sample size calculations	8
Recruitment	15	Strategies for achieving adequate participant enrolment to reach target sample size	4,8
**Methods: Assignment of interventions (for controlled trials)**			
Allocation:			
Sequence generation	16a	Method of generating the allocation sequence (eg, computer-generated random numbers), and list of any factors for stratification. To reduce predictability of a random sequence, details of any planned restriction (eg, blocking) should be provided in a separate document that is unavailable to those who enrol participants or assign interventions	7
Allocation concealment mechanism	16b	Mechanism of implementing the allocation sequence (eg, central telephone; sequentially numbered, opaque, sealed envelopes), describing any steps to conceal the sequence until interventions are assigned	7
Implementation	16c	Who will generate the allocation sequence, who will enrol participants, and who will assign participants to interventions	7
Blinding (masking)	17a	Who will be blinded after assignment to interventions (eg, trial participants, care providers, outcome assessors, data analysts), and how	7
	17b	If blinded, circumstances under which unblinding is permissible, and procedure for revealing a participant's allocated intervention during the trial	NA
**Methods: Data collection, management, and analysis**			

Data collection methods	18a	Plans for assessment and collection of outcome, baseline, and other trial data, including any related processes to promote data quality (eg, duplicate measurements, training of assessors) and a description of study instruments (eg, questionnaires, laboratory tests) along with their reliability and validity, if known. Reference to where data collection forms can be found, if not in the protocol	6
	18b	Plans to promote participant retention and complete follow-up, including list of any outcome data to be collected for participants who discontinue or deviate from intervention protocols	9
Data management	19	Plans for data entry, coding, security, and storage, including any related processes to promote data quality (eg, double data entry; range checks for data values). Reference to where details of data management procedures can be found, if not in the protocol	NA
Statistical methods	20a	Statistical methods for analysing primary and secondary outcomes. Reference to where other details of the statistical analysis plan can be found, if not in the protocol	7-8
	20b	Methods for any additional analyses (eg, subgroup and adjusted analyses)	7-8
	20c	Definition of analysis population relating to protocol non-adherence (eg, as randomised analysis), and any statistical methods to handle missing data (eg, multiple imputation)	7-8
**Methods: Monitoring**			
Data monitoring	21a	Composition of data monitoring committee (DMC); summary of its role and reporting structure; statement of whether it is independent from the sponsor and competing interests; and reference to where further details about its charter can be found, if not in the protocol. Alternatively, an explanation of why a DMC is not needed	NA
	21b	Description of any interim analyses and stopping guidelines, including who will have access to these interim results and make the final decision to terminate the trial	8

For peer review only - http://bmjopen.bmj.com/site/about/guidelines.xhtml

Harms	22	Plans for collecting, assessing, reporting, and managing solicited and spontaneously reported adverse events and other unintended effects of trial interventions or trial conduct	NA
Auditing	23	Frequency and procedures for auditing trial conduct, if any, and whether the process will be independent from investigators and the sponsor	NA
**Ethics and dissemination**			
Research ethics approval	24	Plans for seeking research ethics committee/institutional review board (REC/IRB) approval	9
Protocol amendments	25	Plans for communicating important protocol modifications (eg, changes to eligibility criteria, outcomes, analyses) to relevant parties (eg, investigators, REC/IRBs, trial participants, trial registries, journals, regulators)	NA
Consent or assent	26a	Who will obtain informed consent or assent from potential trial participants or authorised surrogates, and how (see Item 32)	4-5
	26b	Additional consent provisions for collection and use of participant data and biological specimens in ancillary studies, if applicable	NA
Confidentiality	27	How personal information about potential and enrolled participants will be collected, shared, and maintained in order to protect confidentiality before, during, and after the trial	NA
Declaration of interests	28	Financial and other competing interests for principal investigators for the overall trial and each study site	12
Access to data	29	Statement of who will have access to the final trial dataset, and disclosure of contractual agreements that limit such access for investigators	9
Ancillary and post-trial care	30	Provisions, if any, for ancillary and post-trial care, and for compensation to those who suffer harm from trial participation	NA
Dissemination policy	31a	Plans for investigators and sponsor to communicate trial results to participants, healthcare professionals, the public, and other relevant groups (eg, via publication, reporting in results databases, or other data sharing arrangements), including any publication restrictions	9

For peer review only

	31b	Authorship eligibility guidelines and any intended use of professional writers	NA
	31c	Plans, if any, for granting public access to the full protocol, participant-level dataset, and statistical code	9
**Appendices**			
Informed consent materials	32	Model consent form and other related documentation given to participants and authorised surrogates	Appendix A
Biological specimens	33	Plans for collection, laboratory evaluation, and storage of biological specimens for genetic or molecular analysis in the current trial and for future use in ancillary studies, if applicable	NA

*It is strongly recommended that this checklist be read in conjunction with the SPIRIT 2013 Explanation & Elaboration for important clarification on the items. Amendments to the protocol should be tracked and dated. The SPIRIT checklist is copyrighted by the SPIRIT Group under the Creative Commons "Attribution-NonCommercial-NoDerivs 3.0 Unported" license.

For peer review only

BMJ Open

Effects of a waiting list control design on alcohol consumption among online help-seekers: protocol for a randomised controlled trial

Journal:	*BMJ Open*
Manuscript ID	bmjopen-2021-049810.R1
Article Type:	Protocol
Date Submitted by the Author:	27-Jun-2021
Complete List of Authors:	Bendtsen, Marcus; Linköping University<br>Ulfsdotter Gunnarsson, Katarina; Linköping University<br>McCambridge, Jim; University of York, Department of Health Sciences
**Primary Subject Heading**:	Public health
Secondary Subject Heading:	Epidemiology, Ethics
Keywords:	STATISTICS & RESEARCH METHODS, PUBLIC HEALTH, PREVENTIVE MEDICINE

SCHOLARONE™
Manuscripts

I, the Submitting Author has the right to grant and does grant on behalf of all authors of the Work (as defined in the below author licence), an exclusive licence and/or a non-exclusive licence for contributions from authors who are: i) UK Crown employees; ii) where BMJ has agreed a CC-BY licence shall apply, and/or iii) in accordance with the terms applicable for US Federal Government officers or employees acting as part of their official duties; on a worldwide, perpetual, irrevocable, royalty-free basis to BMJ Publishing Group Ltd ("BMJ") its licensees and where the relevant Journal is co-owned by BMJ to the co-owners of the Journal, to publish the Work in this journal and any other BMJ products and to exploit all rights, as set out in our licence.

The Submitting Author accepts and understands that any supply made under these terms is made by BMJ to the Submitting Author unless you are acting as an employee on behalf of your employer or a postgraduate student of an affiliated institution which is paying any applicable article publishing charge ("APC") for Open Access articles. Where the Submitting Author wishes to make the Work available on an Open Access basis (and intends to pay the relevant APC), the terms of reuse of such Open Access shall be governed by a Creative Commons licence – details of these licences and which Creative Commons licence will apply to this Work are set out in our licence referred to above.

Other than as permitted in any relevant BMJ Author's Self Archiving Policies, I confirm this Work has not been accepted for publication elsewhere, is not being considered for publication elsewhere and does not duplicate material already published. I confirm all authors consent to publication of this Work and authorise the granting of this licence.

For peer review only

EFFECTS OF A WAITING LIST CONTROL DESIGN ON ALCOHOL CONSUMPTION AMONG ONLINE HELP-SEEKERS: PROTOCOL FOR A RANDOMISED CONTROLLED TRIAL

Marcus Bendtsen[1,*], Katarina Ulfsdotter Gunnarsson[1], Jim McCambridge[2]

[1] Department of Health, Medicine and Caring Sciences, Linköping University, Sweden.
[2] Department of Health Sciences, University of York.

*Corresponding author
Marcus Bendtsen (marcus.bendtsen@liu.se)
Department of Health, Medicine and Caring Sciences, Division of Society and Health
Linköping University, 581 83 Linköping, registrator@liu.se, +46 13 28 10 00

Word count: 3369

For peer review only

ABSTRACT

Introduction: Sparse attention has been given to the design of control conditions in trials, despite their important role as contrasts for novel treatments, and thus as a key determinant of effect sizes. This undermines valid inferences on effect estimates in trials, which are fundamentally comparative in nature. Such challenges to understanding also makes generalisation of effect estimates complex, for example it may not be clear to what degree real-world alternatives to the novel treatments in pragmatic trials are similar to the control conditions studied. The present study aims to estimate the effects of being allocated to a waiting list control condition.

Methods and analysis: Individuals searching online for help to reduce their drinking will be invited to take part in a study. Individuals aged 18 or older, who in the past month consumed 6 or more drinks on one occasion, or consumed 10 or more drinks the past week, will be eligible to participate. Both groups will receive identical feedback and advice on behaviour change, however, one group will be informed that they have to wait one month for the intervention materials. One month post-randomisation, participants will receive an email with the follow-up questionnaire measuring the primary outcomes: (1) frequency of heavy episodic drinking (defined as at study entry) in the past month; and (2) overall past week alcohol consumption. Differences between groups will be analysed using negative binomial regression models estimated using Bayesian inference. Recruitment will begin in October 2021. A Bayesian group sequential design will be employed to determine when to end enrolment (expected to be between 500 and 1500 individuals).

Ethics and dissemination: The study was approved by the Swedish Ethical Review Authority on 2021-01-25 (Dnr 2020-06267). Findings will be disseminated in open access peer-reviewed journals no later than 2023.

Registration: Prospectively registered on 2021-02-01 (ISRCTN14959594).

ARTICLE SUMMARY

STRENGTHS AND LIMITATIONS OF THIS STUDY

- Sparse attention has been given to the effects of different control conditions.
- Use of a double blind randomised controlled design allows for a valid estimate of the effects of being allocated to a waiting list control.
- The Bayesian group sequential design will allow for recruitment to not last longer than necessary, minimizing the number of participants exposed to the necessary deception involved in the study.
- The trial is an online trial, which usually suffer from high rates of attrition.

For peer review only - http://bmjopen.bmj.com/site/about/guidelines.xhtml

INTRODUCTION

Effects estimated in randomised controlled trials (RCTs) should always be understood as contrasts between allocated conditions [1]. Since effects are contrasts, estimates are affected by either side of the comparison. While much effort is usually placed in designing and describing the novel treatment side of the contrast, typically a fraction of this attention is given to the control side. There is sparse attention to the design of control conditions in trials in the research literature [2–6]. Not only does this potentially introduce bias, and certainly complicates the interpretation of the effects estimated, it also makes generalisability problematic, with implications for research use in decision-making.

To illustrate the complex nature of the generalisability issues, we borrow notation from the potential outcomes framework [7]. Let Y_x represent the outcome for an individual if the individual would hypothetically receive treatment x. There may be a range of different treatments available, and depending on which treatment an individual actually receives, different outcomes will follow. In a 2-arm RCT, the two *potential* outcomes for participants are Y_0 and Y_1 , i.e., the outcomes realised if they are allocated to the control condition ($x = 0$) or the intervention condition ($x = 1$). The individual level causal effect of the intervention is defined as $Y_1 - Y_0$, that is, the potential outcome under the intervention condition minus the potential outcome under the control condition (or some other mode of contrast, e.g. risk or odds). Of course, when using between-subjects designs it will be impossible to know both Y_1 and Y_0 for the same individual, thus RCTs are generally focused on the population level causal effect. With continuous outcomes, this is commonly the expected difference $E[Y_1] - E[Y_0]$ between the two conditions. We rely on the license given to us by virtue of randomisation to claim that these are unconfounded estimates with sufficiently large numbers, protecting the internal validity of our estimates.

Ideally, $E[Y_1] - E[Y_0]$ would be an estimate which arises from an effectiveness or pragmatic trial rather than an efficacy or explanatory trial [8,9], which can be interpreted as the effect anticipated in a real-world roll-out. Y_1 could be a novel potential outcome which we may offer to individuals, and it may be close to what was estimated in the trial if the study inclusion criteria were similar to routine practice, there were no marked bias, and if treatment protocols are followed. However, a problem arises if Y_0 is not the potential outcome which would be the alternative to Y_1 in the real world. For instance, if Y_0 represents the potential outcome of being placed on a waiting list [10], then that is not an outcome which is available to individuals in a real-world setting, except in those situations where there are waiting lists for treatment. To avoid any such problem the control condition in the trial needs to be identical to usual care. If being placed on a waiting list in a study affects individuals in any way differently than do the real-world alternatives to Y_1, then the external validity of the contrast $E[Y_1] - E[Y_0]$ should be questioned, and measures need to be taken to facilitate the interpretation of the observed effect.

WAITING LIST CONTROLS

Rather than withholding treatment from controls in RCTs altogether, it has been argued that it is more ethical to offer the treatment to controls after the trial period [10] – i.e. placing participants on a waiting list. This type of control condition is commonly used in behavioural and rehabilitation treatments [11], altering the nature of Y_0. It is not well understood how individuals react to these decisions. For example, in one smoking cessation trial some control participants had negative feelings about having to wait, and decided to quit later [12]. Researchers have long been concerned with feelings of disappointment about allocation to control conditions in trials more broadly, but we know little about how study participants subsequently act on them [13,14]. This may be especially the case in online studies when participants actively engage with recruitment procedures and may therefore have higher expectations of receiving novel treatment, rather than simply respond to in-person contacts from researchers that they have not initiated. On the other hand, the effects of being

For peer review only - http://bmjopen.bmj.com/site/about/guidelines.xhtml

informed that one has to wait, as investigated here, may be less pronounced in online studies [15–19]. It therefore remains unclear if these reactions translate into any markedly biased effect estimates.

Indirect evidence suggests that in many circumstances we may expect participants in control conditions to change more than they ordinarily would if not involved in a study. It has for instance been found that alcohol consumption tends to decrease in control groups by approximately 20% in alcohol intervention trials [20–22]. This change is likely due to a combination of several factors, including regression to the mean [23], assessment effects [24], and other forms of research participation effects [25–27]. A network meta-analysis of cognitive behaviour therapy for depression [28], found in contrast that participants in waiting list control groups improved less than expected. This is in line with thinking that when patients are placed on waiting lists, they are to some extent compliant with the implicit direction; they wait, and avoid instigating changes [29].

A direct estimation of the effects of a waiting list control design on alcohol consumption was conducted among 185 hazardous or harmful drinkers recruited through newspaper advertisements in Canada [30]. A 2-arm parallel groups RCT was employed, in which both groups received the same brief alcohol intervention, but one group were told that they had been allocated to the intervention arm, and the other told they were allocated to a waiting list arm. This exploratory trial found an interaction between being allocated to the waiting list group and the action subscale of the readiness to change questionnaire [31,32]. This suggested that those in the waiting list group who rated themselves above the median on the action subscale (thus being more ready for action) reported consuming more alcohol in comparison to their counterparts in the intervention group (approximately 6 drinks more per week and 1.3 more drinks on the occasion when they drank the most). They thus waited to reduce their drinking. While findings from the trial were mixed, it generated hypotheses important to study further. The current trial described in this protocol will address these hypotheses with a larger sample size in an online recruitment setting.

OBJECTIVES

Using a 2-arm (1:1) randomised controlled trial, the objective of this study is to estimate the effects of being allocated to a waiting list control group on alcohol consumption. The primary effects being estimated are the total effect and moderation by readiness to change. We anticipate that these effects will be in the direction of higher consumption at follow-up among waiting list participants in comparison to non-waiting list participants.

METHODS AND ANALYSIS

This protocol contains relevant items from the Standard Protocol Items: Recommendations for Interventional Trials (SPIRIT) [33]. The trial has been prospectively registered in the ISRCTN registry on 2021-02-01 (ISRCTN14959594), and has received ethical approval by the Swedish Ethical Review Authority on 2021-01-25 (Dnr 2020-06267).

STUDY SETTING, ELIGIBILITY CRITERIA AND RECRUITMENT

Participants will be recruited through online advertisements (Google Ads), using language targeting to include individuals using Google's services in English (including search and gmail). Keywords will be selected to target those seeking help to drink less, e.g. "How do I drink less", "I drink too much", and "Support for drinkers". The adverts will be designed to target individuals who are willing to take part in a trial of a self-help alcohol intervention. An example advert is shown in Figure 1, and study information presented to individuals who click on the advert can be found in Appendix A. Recruitment will begin in October 2021 and last for no more than 24 months (October 2023, see Sample Size).

INSERT FIGURE1 HERE

For peer review only - http://bmjopen.bmj.com/site/about/guidelines.xhtml

Figure 1 - Example advert shown to individuals who search online for help to drink less

Individuals who consent to take part in the trial will be asked to complete a baseline questionnaire (see Appendix B). Individuals will first be asked to state their age and sex, the number of times during the past month they consumed 6 or more drinks on one occasion, and the number of drinks consumed in the past week. Individuals will be included if they report having consumed 6 or more drinks on one occasion at least once in the past month or having consumed more than 9 drinks in the past week. Individuals younger than 18 years old will be excluded. Those not excluded will be asked to complete the readiness to change questionnaire. After completing the questionnaire, individuals will be asked to leave an email address to which further information about study participation will be sent. The email will be sent immediately and will include a brief text explaining that a link should be clicked in the email to continue participation in the study. Individuals who click on the link in the email will be randomised to either the intervention group (IG) or the waiting list control group (WL).

INTERVENTIONS

Participants in both groups (IG and WL) will receive the same personalised feedback and advice based on the baseline questionnaire. The material, which has been developed from our previous research [24,34–40] and is based around social cognitive theories of health behaviour [41], includes: (1) feedback on current consumption in relation to recommendations, (2) content to increase motivation and self-efficacy, and (3) advice on what to do to reduce drinking and avoid environmental triggers.

Preceding the feedback and advice will be a page with study information which will be different between groups; this is the experimental contrast in the present study.

Text to IG group
There are two groups in this study. You are in the group which receives immediate feedback and support tailored to the responses to the questionnaire you previously completed. This material has been designed for people who are concerned about their drinking. [Button: Take me to the feedback].

Text to WL group
There are two groups in this study. You are in the group which will have to wait for the feedback and support material. We will contact you again in four weeks, and at that time you will be given the material. In the meantime, we have generated a report based on your responses to the questionnaire previously completed. [Button: Take me to the feedback].

OUTCOMES

Primary:

1. Frequency of heavy episodic drinking in the past month
2. Overall consumption the past week

Secondary:

3. Readiness to change

The two primary outcomes have been selected to be similar to the outcomes used in the exploratory trial upon which this trial has been modelled [30], while at the same time being part of the proposed core outcome set for brief alcohol interventions [42,43]. Frequency of heavy episodic drinking will be defined as the number of times in the past month the participant has consumed 6 or more drinks on one occasion. Number of drinks consumed in the past week will be assessed by asking participants to report their consumption day-by-day.

For peer review only - http://bmjopen.bmj.com/site/about/guidelines.xhtml

The secondary outcome, readiness to change, will be measured using the readiness to change questionnaire (treatment version) [31,32]. While our primary use of this scale is for assessing moderation effects (using the baseline assessment), we are also interested in estimating effects of the WL condition on readiness to change directly.

FOLLOW-UP

One month post randomisation, participants will be sent an email including a link to the follow-up questionnaire (see Appendix B). Participants will be sent two reminders three days apart. If no response has been recorded three days after the final reminder, a fourth email will be sent with the frequency of heavy episodic drinking in the past month measure embedded in the email. Reminders will only be sent to participants who have not yet responded to the two primary alcohol consumption outcomes. Participants in the WL group will immediately after completing the outcome measure be sent an email with information about the deception involved in the study (Appendix C). A participant timeline is shown in Figure 2.

INSERT FIGURE2 HERE

Figure 2 - Participant timeline (SPIRIT figure)

ALLOCATION AND BLINDING

Block randomisation will be used with randomly permuted block sizes of 2 and 4 to ensure equal size groups. Randomisation will be automatic and computerised on the backend server, thus neither research personnel nor participants will be able to influence allocation. Participants will be blind to the true nature of the study. Research personnel will be blind to allocation.

STATISTICAL METHODS

All analyses will be conducted according to intention-to-treat principles, with all participants analysed in the groups to which they were randomised. Analyses will be done using available data with missing data imputed. Attrition analyses will explore the plausibility of the implicit missing at random (MAR) assumption underlying the analyses. Model parameters will be interpreted by inspecting marginal posterior distributions using Bayesian inference (see Sample Size for priors) [44], and imputation will be done using multiple imputation with chained equations. We will use R version 3.6 with the RStan library for all analyses [45].

PRIMARY ANALYSES

The analysis of primary and secondary outcomes will be conducted through regression models in which each outcome will be regressed against group allocation. One regression model per outcome will be created. Negative binomial regression will be used for both primary outcomes and linear regression will be used for readiness to change scores (precontemplation, contemplation and action). A multinomial regression model will also be estimated for stage of change designation (i.e. highest arithmetical score among the three sub scales with ties preferring the stage farther along the continuum of change [31,32]). All regression models will be adjusted for age, sex and each respective outcome measured at baseline.

EXPLORATORY ANALYSES

Effect modification analyses of group allocation and readiness to change scores (precontemplation, contemplation and action) on the primary outcomes will be analysed by adding interaction terms to the respective regression models. We will estimate separate models for each stage interaction, and a combined

For peer review only - http://bmjopen.bmj.com/site/about/guidelines.xhtml

model with all stages interacting with allocation using shrinkage priors [46]. We will also explore effect modification with respect to group allocation and stage of change designation on the primary outcomes.

Separate analyses of the heavy episodic drinking outcome (first primary outcome) will be conducted among those who at baseline had consumed 6 or more drinks on one or more occasions over the past month – thus excluding those who had no episodes of heavy drinking the month preceding enrolment. Similarly, separate analyses of the overall consumption the past week (second primary outcome) will be conducted among those who at baseline reported being at-risk with respect to weekly consumption – thus excluding those who at baseline reported having consumed 9 drinks or fewer the past week.

MEDIATOR ANALYSES

We will estimate the degree to which the effect of allocation on the primary outcomes is mediated through two variables: (1) accessing the intervention materials and (2) time spent on the intervention materials. We will use a causal inference framework [47–49], using Bayesian inference to estimate the natural direct effect and natural indirect effect (as per the definitions of Pearl [49]). We will report on the posterior distributions of these two estimates, as well as the proportion of the total effect which is accounted for by the natural indirect effect. We will also explore if readiness to change moderates any of these effects.

ATTRITION ANALYSES

The available data and imputation approach used in the primary analyses may result in biased intention-to-treat estimates if data is not MAR. We will therefore seek evidence against the MAR assumption in two attrition analyses. First, since late responders to follow-up may be more alike non-responders than early responders are, we will investigate if outcomes are systematically different given the number of reminders needed to collect follow-up data. Second, we will explore if responders and non-responders are systematically different with respect to baseline characteristics, using logistic regression models with shrinkage priors.

SAMPLE SIZE

The trial will use a Bayesian group sequential design to monitor recruitment with interim analyses planned for every 25 participants completing the follow-up questionnaire [50–52]. The primary outcomes will be modelled following the analysis plan presented earlier, and each covariate representing group allocation will be assessed for evidence of effect or futility. Covariates representing the interaction between the stages of readiness for change and group allocation will also be monitored (using separate models for each stage interaction). Let β_i represent the covariates of interest, and D the data available, then for each β_i the target criteria will be:

- **Effect**: $p(\beta_i > 0|D) > 97.5\%$ or $p(\beta_i < 0|D) > 97.5\%$ (i.e. the effect is greater (or less) than 0 with a probability greater than 97.5%).

- **Futility**: $p(\log(\frac{1}{1.10}) < \beta_i < \log(1.10)|D) > 95.0\%$ (i.e. the incidence rate ratio is greater than 1/1.10 and less than 1.10 with a probability greater than 95%).

For the effect criterion, we will use a sceptical normal prior for covariates (mean = 0 and standard deviation = 1.0), and a wider prior will be used for the futility criterion (mean = 0 and standard deviation = 2.0).

The criteria above should be viewed as targets, thus at each interim analysis we will evaluate each criterion for each covariate and make a decision if we believe that recruitment should end. Simulations indicate that we will require a sample size in the range of 500 to 1500 participants. Recruitment will not exceed 24 months.

For peer review only - http://bmjopen.bmj.com/site/about/guidelines.xhtml

PATIENT AND PUBLIC INVOLVEMENT STATEMENT

Patients were not involved in the planning of this study.

DISCUSSION

Trials are usually advertised as important for research, but they may also be seen as an opportunity for prospective participants to get access to novel treatments. Participants have likely found the trial, and enrolled, motivated by the ways in which the trial may benefit them. However, trials do not primarily advertise the conditions which lead to Y_0, i.e., recruitment material never says “Do you want to be on a waiting list?”. The unintended consequences of telling participants that they will have to wait is the motivation for the present study, which is designed to uncover some of these consequences in the chosen setting.

The findings from the present study will help to understand if participants are affected by being told that they are being placed in a waiting list control group and will therefore help to guide decisions of comparator choice in future studies of brief alcohol interventions. In addition, effect estimates from the study will also support the possibility of retrospectively adjusting findings from previous trials of brief alcohol interventions to account for any bias induced by waiting list controls [53].

LIMITATIONS

Online studies of alcohol interventions commonly suffer from high levels of attrition, particularly at follow-up, and we expect the primary limitation of this study to be high attrition rates. To reduce attrition, we will only randomise participants after they have confirmed their email address, which helps to ensure that we have a valid email address for each randomised participant. We will use reminders over a short time span to keep participants aware that it is important that they respond, and finally make it easy for them to respond by including one of the primary outcome measures in the body of the email. The Bayesian group sequential design will ensure that we collect data for as long as is necessary to fulfil the criteria (see Sample Size), which will ensure that we have enough samples for our analyses. However, this will not protect against differential attrition, and thus does not protect against attrition bias, so we will need to study this risk carefully.

ETHICS AND DISSEMINATION

The main ethical concern in this trial is the use of deception - required to not reveal to prospective participants that it is the effects of allocation which are being studied. Routine use of deception in research is not recommended, however, considering the widespread use of waiting list designs, understanding their effects was considered important and justified the risks induced by deception. The study was approved by the Swedish Ethical Review Authority on 2021-01-25 (Dnr 2020-06267). Data collection will begin in October 2021 and last no longer than 24 months. The dataset will be made available to researchers upon reasonable request. Findings will be published in peer-reviewed journals and presented at relevant conferences no later than 2023.

REFERENCES

1. Barkauskas VH, Lusk SL, Eakin BL. Selecting Control Interventions for Clinical Outcome Studies. West J Nurs Res. 2005 Apr;27(3):346–63.

2. Cook TD, Campbell DT. Quasi-Experimentation: Design and Analysis Issues for Field Settings. Houghton Mifflin; 1979.

For peer review only

3. Rosnow RL, Rosenthal R. People studying people: Artifacts and ethics in behavioral research. New York: W.H. Freeman; 1997.

4. Freedland KE, King AC, Ambrosius WT, Mayo-Wilson E, Mohr DC, Czajkowski SM, et al. The selection of comparators for randomized controlled trials of health-related behavioral interventions: recommendations of an NIH expert panel. J Clin Epidemiol. 2019 Jun;110:74–81.

5. Freedland KE. Demanding Attention: reconsidering the role of attention control groups in behavioral intervention research. Psychosom Med. 2013;75(2):100–2.

6. Freedland KE, Mohr DC, Davidson KW, Schwartz JE. Usual and Unusual Care: existing practice control groups in randomized controlled trials of behavioral interventions. Psychosom Med. 2011 May;73(4):323–35.

7. Rubin DB. Estimating causal effects of treatments in randomized and nonrandomized studies. J Educ Psychol. 1974;66(5):688–701.

8. Singal AG, Higgins PDR, Waljee AK. A Primer on Effectiveness and Efficacy Trials. Clin Transl Gastroenterol. 2014 Jan;5(1):e45.

9. Roland M, Torgerson DJ. Understanding controlled trials: What are pragmatic trials? BMJ. 1998 Jan 24;316(7127):285–285.

10. Elliott SA, Brown JSL. What are we doing to waiting list controls? Behav Res Ther. 2002 Sep;40(9):1047–52.

11. Hart T, Bagiella E. Design and Implementation of Clinical Trials in Rehabilitation Research. Arch Phys Med Rehabil. 2012 Aug;93(8):S117–26.

12. Müssener U, Linderoth C, Bendtsen M. Exploring the Experiences of Individuals Allocated to a Control Setting: Findings From a Mobile Health Smoking Cessation Trial. JMIR Hum Factors. 2019;6(2):e12139.

13. McCambridge J, Sorhaindo A, Quirk A, Nanchahal K. Patient preferences and performance bias in a weight loss trial with a usual care arm. Patient Educ Couns. 2014 May;95(2):243–7.

14. Howick J. Questioning the Methodologic Superiority of 'Placebo' Over 'Active' Controlled Trials. Am J Bioeth. 2009 Sep 11;9(9):34–48.

15. McCambridge J, Wilson A, Attia J, Weaver N, Kypri K. Randomized trial seeking to induce the Hawthorne effect found no evidence for any effect on self-reported alcohol consumption online. J Clin Epidemiol. 2019 Apr;108:102–9.

16. Kypri K, Wilson A, Attia J, Sheeran P, Miller P, McCambridge J. Social Desirability Bias in the Reporting of Alcohol Consumption: A Randomized Trial. J Stud Alcohol Drugs. 2016 May;77(3):526–31.

17. McCambridge J. From question-behaviour effects in trials to the social psychology of research participation. Psychol Health. 2015 Jan 2;30(1):72–84.

18. Kypri K, Wilson A, Attia J, Sheeran PJ, McCambridge J. Effects of study design and allocation on self-reported alcohol consumption: randomized trial. Trials. 2015 Dec;16(1):127.

19. Kypri K, Bowe SJ, Karlsson N, McCambridge J. Enrolment-latency in randomized behavior change trials: individual participant data meta-analysis showed association with attrition but not effect-size. J Clin Epidemiol. 2020 Feb;118:55–9.

For peer review only

For peer review only - http://bmjopen.bmj.com/site/about/guidelines.xhtml

20. Jenkins RJ, McAlaney J, McCambridge J. Change over time in alcohol consumption in control groups in brief intervention studies: systematic review and meta-regression study. Drug Alcohol Depend. 2009 Feb;100(1–2):107–14.

21. Jenkins RJ, McAlaney J, McCambridge J. Corrigendum to “Change over time in alcohol consumption in control groups in brief intervention studies: Systematic review and meta-regression study” [Drug Alcohol Depend. 100 (2009) 107–114]. Drug Alcohol Depend. 2010 Apr;108(1–2):151.

22. Moyer A, Finney JW. Outcomes for untreated individuals involved in randomized trials of alcohol treatment. J Subst Abuse Treat. 2002 Oct;23(3):247–52.

23. Linden A. Assessing regression to the mean effects in health care initiatives. BMC Med Res Methodol. 2013 Dec 28;13(1):119.

24. McCambridge J, Bendtsen M, Karlsson N, White IR, Nilsen P, Bendtsen P. Alcohol assessment and feedback by email for university students: Main findings from a randomised controlled trial. Br J Psychiatry. 2013;203(5):334–40.

25. McCambridge J, Witton J, Elbourne DR. Systematic review of the Hawthorne effect: New concepts are needed to study research participation effects. J Clin Epidemiol. 2014 Mar;67(3):267–77.

26. McCambridge J, Kypri K, Elbourne D. Research participation effects: a skeleton in the methodological cupboard. J Clin Epidemiol. 2014 Aug;67(8):845–9.

27. McCambridge J, Kypri K, McElduff P. Regression to the mean and alcohol consumption: A cohort study exploring implications for the interpretation of change in control groups in brief intervention trials. Drug Alcohol Depend. 2014 Feb;135:156–9.

28. Furukawa TA, Noma H, Caldwell DM, Honyashiki M, Shinohara K, Imai H, et al. Waiting list may be a nocebo condition in psychotherapy trials: a contribution from network meta-analysis. Acta Psychiatr Scand. 2014 Sep;130(3):181–92.

29. Miller W, Rollnick S. Motivational Interviewing: Preparing People for Change. New York: Guilford Press; 2002.

30. Cunningham JA, Kypri K, McCambridge J. Exploratory randomized controlled trial evaluating the impact of a waiting list control design. BMC Med Res Methodol. 2013 Dec;13(1):150.

31. Rollnick S, Heather N, Gold R, Hall W. Development of a short ‘readiness to change’ questionnaire for use in brief, opportunistic interventions among excessive drinkers. Addiction. 1992 May;87(5):743–54.

32. Heather N, Hönekopp J. A revised edition of the Readiness to Change Questionnaire [Treatment Version]. Addict Res Theory. 2008 Jan 11;16(5):421–33.

33. Chan A-W, Tetzlaff JM, Altman DG, Laupacis A, Gøtzsche PC, Krleža-Jerić K, et al. SPIRIT 2013 Statement: Defining Standard Protocol Items for Clinical Trials. Ann Intern Med. 2013 Feb 5;158(3):200.

34. Bendtsen P, McCambridge J, Bendtsen M, Karlsson N, Nilsen P. Effectiveness of a Proactive Mail-Based Alcohol Internet Intervention for University Students: Dismantling the Assessment and Feedback Components in a Randomized Controlled Trial. J Med Internet Res. 2012 Oct 31;14(5):e142.

35. McCambridge J, Bendtsen P, Bendtsen M, Nilsen P. Alcohol email assessment and feedback study dismantling effectiveness for university students (AMADEUS-1): study protocol for a randomized controlled trial. Trials. 2012 Dec 6;13(1):49.

For peer review only

36. McCambridge J, Bendtsen M, Karlsson N, White IR, Bendtsen P. Alcohol assessment & feedback by e-mail for university student hazardous and harmful drinkers: Study protocol for the AMADEUS-2 randomised controlled trial. BMC Public Health. 2013;13(1):949.

37. Bendtsen P, Bendtsen M, Karlsson N, White IR, McCambridge J. Online Alcohol Assessment and Feedback for Hazardous and Harmful Drinkers: Findings From the AMADEUS-2 Randomized Controlled Trial of Routine Practice in Swedish Universities. J Med Internet Res. 2015;17(7):e170.

38. Bendtsen M. An Electronic Screening and Brief Intervention for Hazardous and Harmful Drinking Among Swedish University Students: Reanalysis of Findings From a Randomized Controlled Trial Using a Bayesian Framework. J Med Internet Res. 2019;21(12):e14420.

39. Bendtsen M. Electronic Screening for Alcohol Use and Brief Intervention by Email for University Students: Reanalysis of Findings From a Randomized Controlled Trial Using a Bayesian Framework. J Med Internet Res. 2019;21(11):e14419.

40. Bendtsen M, McCambridge J. Reducing Alcohol Consumption Among Risky Drinkers in the General Population of Sweden Using an Interactive Mobile Health Intervention: Protocol for a Randomized Controlled Trial. JMIR Res Protoc. 2019;8(4):e13119.

41. Conner M, Norman P. Predicting Health Behavior: Research and Practice with Social Cognition Models. 2005.

42. Shorter GW, Bray JW, Giles EL, O'Donnell AJ, Berman AH, Holloway A, et al. The Variability of Outcomes Used in Efficacy and Effectiveness Trials of Alcohol Brief Interventions: A Systematic Review. J Stud Alcohol Drugs. 2019 May;80(3):286–98.

43. Shorter GW, Heather N, Bray JW, Berman AH, Giles EL, O'Donnell AJ, et al. Prioritization of Outcomes in Efficacy and Effectiveness of Alcohol Brief Intervention Trials: International Multi-Stakeholder e-Delphi Consensus Study to Inform a Core Outcome Set. J Stud Alcohol Drugs. 2019 May;80(3):299–309.

44. Bendtsen M. A Gentle Introduction to the Comparison Between Null Hypothesis Testing and Bayesian Analysis: Reanalysis of Two Randomized Controlled Trials. J Med Internet Res. 2018;20(10):e10873.

45. Team SD. RStan: the R interface to Stan. 2020.

46. Piironen J, Vehtari A. Sparsity information and regularization in the horseshoe and other shrinkage priors. Electron J Stat. 2017;11(2):5018–51.

47. Imai K, Keele L, Tingley D. A General Approach to Causal Mediation Analysis. Psychol Methods. 2010;15(4):309–34.

48. Pearl J. Causality. Causality: Models, Reasoning, and Inference, Second Edition. Cambridge: Cambridge University Press; 2009.

49. Pearl J. Interpretation and identification of causal mediation. Psychol Methods. 2014;19(4):459–81.

50. Gsponer T, Gerber F, Bornkamp B, Ohlssen D, Vandemeulebroecke M, Schmidli H. A practical guide to Bayesian group sequential designs. Pharm Stat. 2014;13(1):71–80.

51. Berry DA. Bayesian clinical trials. Nat Rev Drug Discov. 2006 Jan;5(1):27–36.

52. Bendtsen M. The P Value Line Dance: When Does the Music Stop? J Med Internet Res. 2020;22(8):e21345.

53. Bendtsen M, McCambridge J. Causal models accounted for research participation effects when estimating effects in a behavioral intervention trial. J Clin Epidemiol. 2021 Mar 13;136:77–83.

For peer review only

AUTHORS' CONTRIBUTIONS

MB and JM defined the study objectives and designed the trial. All authors were involved in developing the intervention materials. MB and KUG are responsible for data collection and statistical analysis. All authors are involved in interpreting findings and all authors read and approved the protocol manuscript.

FUNDING

This research received no specific grant from any funding agency in the public, commercial or not-for-profit sectors.

COMPETING INTERESTS

MB owns a private company (Alexit AB) which disseminates digital lifestyle interventions such as the one offered to participants in this trial. Alexit AB had no part in funding, planning, or execution of this study.

DATA AVAILABILITY STATEMENT

The dataset will be made available to researchers upon reasonable request.

FIGURE LEGENDS

Figure 3 - Example advert shown to individuals who search online for help to drink less

Figure 2 - Participant timeline (SPIRIT figure)

For peer review only

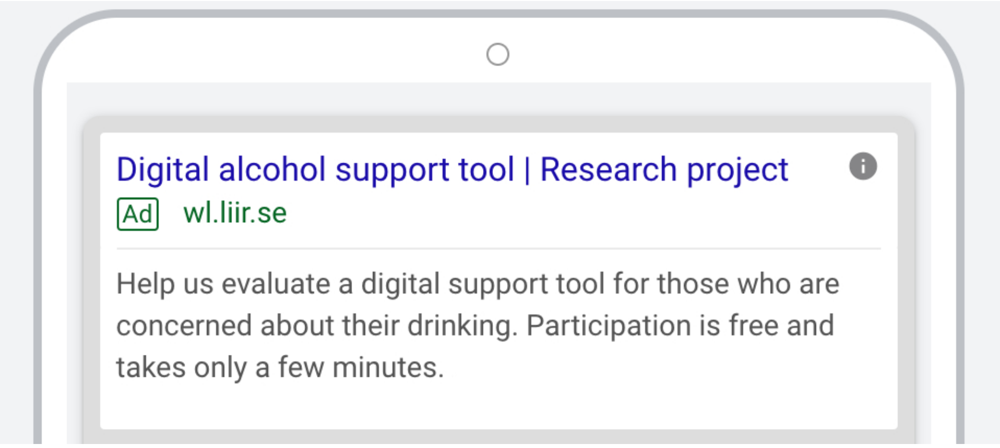

Example advert shown to individuals who search online for help to drink less

	STUDY PERIOD			
	**Enrolment**	**Allocation**	**Post-allocation**	**Close-out**
**TIMEPOINT**	**0**	**0**	***Access to feedback and support material***	***1-month***
**ENROLMENT:**				
Informed consent	X			
Eligibility screen	X			
Allocation		X		
Debriefing				X
**INTERVENTIONS:**				
Intervention group (IG)			↔	
Waiting list group (WL)			↔	
**ASSESSMENTS:**				
Baseline questionnaire	X			
Follow-up questionnaire				X
Accessing material			X	X
Time spent on material			X	

For peer review only - http://bmjopen.bmj.com/site/about/guidelines.xhtml

For peer review only

APPENDIX A – INFORMED CONSENT MATERIALS

When clicking on the online advert, participants will be shown the following information about the study.

<u>Do you want to participate in the "Digital support for alcohol" study?</u>

Thank you for showing interest in this study. By pressing the button "I consent to take part in this study" you are consenting to take part in the study described below.

Why are we doing this study and why do you want me to participate? We have developed a digital support tool which is tailored to individual responses to a questionnaire. The tool has been designed for people who are concerned about their drinking. In order to evaluate if the tool is effective, we are asking people who search online to be part of this study.

This research project is conducted at Linköping university in Sweden, please find contact details for the primary investigator below.

What is expected of me? If you take part in the study you will first be asked to respond to a 5 minute questionnaire. Thereafter you will be asked to leave an email address to which we can send more information about the study. You will be randomly assigned to either get direct access to the tool or wait one month before being given access. One month after having signed up for the study we will send an email to you with a link to a short follow-up questionnaire.

Will I be taking any risks by participating in this study? Participating in this study will give you access to a novel digital support tool which has been designed to help people concerned about their drinking. However, you may find that the support given to you is inadequate and does not help you, and you may be de-motivated by this. You should be aware of this before deciding to take part in this study. We are however not asking you to refrain from any other support which may be available to you. For instance, you may find the information available at this website helpful: Rethinking Drinking (https://www.rethinkingdrinking.niaaa.nih.gov/).

Will I be leaving any personal information? We will collect data from you about your age, biological sex, alcohol consumption, and your readiness to change. We will also collect an email address from you so that we can contact you for follow-up one month after enrolling. The data collection is for scientific research, and is therefore motivated by public interest (GDPR EU 2016/679, Prop. 2017/18:298). Your data will be treated with confidentiality and we will take measures to ensure that no unauthorized access is granted.

Linköping University will be responsible for your personal data. According to EU law, you retain the right to, without cost, be given access to your personal data and request that changes be made. You can also request that your personal data be deleted. Your rights for deletion of data is however restricted if the data is deemed necessary for the study to be conducted. If you want access to your personal data you should contact the primary investigator Marcus Bendtsen (see information below). The data protection officer can be contacted at dataskyddsombud@liu.se. If you are not satisfied with how your personal data has been handled, you can make a formal complain to the Swedish Authority for Privacy Protection.

Where will data be stored and who will have access? During the study period we will store all data on a database at Linköping university in Sweden. After the study period the data will be archived securely at Linköping university and removed from the database. Access to the data will at first be restricted to the research team, but will later be made available for other researchers by request. Please note that once the study period is complete, all email addresses will be deleted and data will therefore be anonymous.

Who is responsible for the study? Linköping university is responsible for the data collected in this study. If you have any questions about your data you can contact Dr. Marcus Bendtsen, (marcus.bendtsen@liu.se), or the data protection officer (dataskyddsombudet@liu.se). If you are not satisfied with the way your personal data has been handled, you can file a complaint at the Swedish Data Protection Authority.

How can I get more information about the results from this study? All results will be published in peer-reviewed journals and conferences no later than 2022.

Participation is voluntary. You can at any point decide to not be part of the study and you will not have to explain why.

For peer review only

Contact details

Dr. Marcus Bendtsen, marcus.bendtsen@liu.se, 013-28 69 75
Docent in Medical Informatics
Senior Lecturer in Experimental Social Medicine and Public Health
Department of Health, Medicine and Caring Sciences, Linköping University
581 83 Linköping, Sweden

For peer review only

APPENDIX B - QUESTIONNAIRES

BASELINE AND FOLLOW-UP

1. (Baseline only) How old are you? (numeric)

2. (Baseline only) To assess risks from alcohol consumption, we need to know your biological sex?
    a. Female
    b. Male

3. In the past month, how often did you have six or more drinks on one occasion? (numeric)

4. Thinking about the past week, how many standard drinks did you have on: (numeric)
    a. Monday
    b. Tuesday
    c. Wednesday
    d. Thursday
    e. Friday
    f. Saturday
    g. Sunday

Note: A visual guide will be presented to participants with the definition of a standard drink.

The following questions are designed to identify how you personally feel about your drinking right now. Please think about your current situation and drinking habits, even if you have given up drinking completely. Read each question below carefully and then decide whether you agree or disagree with the statements.

Note: The options for each question below are Strongly disagree, Disagree, Unsure, Agree, Strongly agree. The letter in the parenthesis after the question indicates which stage of change the item belongs to and will not be shown to participants (PC = Precontemplation, C = Contemplation, A = Action).

5. It is a waste of time thinking about my drinking because I do not have a problem. (PC)
6. I enjoy my drinking but sometimes I drink too much. (C)
7. There is nothing seriously wrong with my drinking. (PC)
8. Sometimes I think I should quit or cut down my drinking. (C)
9. Anyone can talk about wanting to do something about their drinking, but I'm actually doing something about it. (A)
10. I am a fairly normal drinker. (PC)
11. My drinking is a problem sometimes. (C)
12. I am actually changing my drinking habits right now (either cutting down or quitting). (A)
13. I have started to carry out a plan to cut down or quit drinking. (A)
14. There is nothing I really need to change about my drinking. (PC)
15. Sometimes I wonder if my drinking is out of control. (C)
16. I am actively working on my drinking problem. (C)

For peer review only

APPENDIX C – DEBRIEFING EMAIL

The following email will be sent to the waiting list group after study completion.

Thank you for taking part in the “Digital support for alcohol” study.

The point of the study was to explore if being told to wait for the alcohol support tool made a difference to your alcohol consumption. Therefore, the material you received when you signed up for the study was the support tool which the other group also received. They were however told that they were given the support tool immediately.

This research is important because many studies use a design where participants are asked to wait before they get a new treatment, and it is uncertain what the effects of being told to wait are.

If you have questions about the study and its design, you are welcome to contact the primary investigator Marcus Bendtsen (see below for details).

If you want more support for change of your alcohol consumption you may find the information on this website helpful: Rethinking Drinking (https://www.rethinkingdrinking.niaaa.nih.gov/).

Contact details
Dr. Marcus Bendtsen, marcus.bendtsen@liu.se, 013-28 69 75
Docent in Medical Informatics
Senior Lecturer in Experimental Social Medicine and Public Health
Department of Health, Medicine and Caring Sciences, Linköping University
581 83 Linköping, Sweden

For peer review only

SPIRIT
STANDARD PROTOCOL ITEMS: RECOMMENDATIONS FOR INTERVENTIONAL TRIALS

SPIRIT 2013 Checklist: Recommended items to address in a clinical trial protocol and related documents*

Section/item	Item No	Description	Page Number on which item is reported
**Administrative information**			
Title	1	Descriptive title identifying the study design, population, interventions, and, if applicable, trial acronym	1
Trial registration	2a	Trial identifier and registry name. If not yet registered, name of intended registry	2
	2b	All items from the World Health Organization Trial Registration Data Set	2
Protocol version	3	Date and version identifier	NA
Funding	4	Sources and types of financial, material, and other support	2,12
Roles and responsibilitie s	5a	Names, affiliations, and roles of protocol contributors	1
	5b	Name and contact information for the trial sponsor	NA
	5c	Role of study sponsor and funders, if any, in study design; collection, management, analysis, and interpretation of data; writing of the report; and the decision to submit the report for publication, including whether they will have ultimate authority over any of these activities	NA
	5d	Composition, roles, and responsibilities of the coordinating centre, steering committee, endpoint adjudication committee, data management team, and other individuals or groups overseeing the trial, if applicable (see Item 21a for data monitoring committee)	NA
**Introduction**			

For peer review only - http://bmjopen.bmj.com/site/about/guidelines.xhtml

Background and rationale	6a	Description of research question and justification for undertaking the trial, including summary of relevant studies (published and unpublished) examining benefits and harms for each intervention	3-4
	6b	Explanation for choice of comparators	3-4
Objectives	7	Specific objectives or hypotheses	4
Trial design	8	Description of trial design including type of trial (eg, parallel group, crossover, factorial, single group), allocation ratio, and framework (eg, superiority, equivalence, noninferiority, exploratory)	4
**Methods: Participants, interventions, and outcomes**			
Study setting	9	Description of study settings (eg, community clinic, academic hospital) and list of countries where data will be collected. Reference to where list of study sites can be obtained	4-5
Eligibility criteria	10	Inclusion and exclusion criteria for participants. If applicable, eligibility criteria for study centres and individuals who will perform the interventions (eg, surgeons, psychotherapists)	4-5
Interventions	11a	Interventions for each group with sufficient detail to allow replication, including how and when they will be administered	5
	11b	Criteria for discontinuing or modifying allocated interventions for a given trial participant (eg, drug dose change in response to harms, participant request, or improving/worsening disease)	NA
	11c	Strategies to improve adherence to intervention protocols, and any procedures for monitoring adherence (eg, drug tablet return, laboratory tests)	4-5
	11d	Relevant concomitant care and interventions that are permitted or prohibited during the trial	NA
Outcomes	12	Primary, secondary, and other outcomes, including the specific measurement variable (eg, systolic blood pressure), analysis metric (eg, change from baseline, final value, time to event), method of aggregation (eg, median, proportion), and time point for each outcome. Explanation of the clinical relevance of chosen efficacy and harm outcomes is strongly recommended	6

For peer review only - http://bmjopen.bmj.com/site/about/guidelines.xhtml

Participant timeline	13	Time schedule of enrolment, interventions (including any run-ins and washouts), assessments, and visits for participants. A schematic diagram is highly recommended (see Figure)	Figure 2
Sample size	14	Estimated number of participants needed to achieve study objectives and how it was determined, including clinical and statistical assumptions supporting any sample size calculations	8
Recruitment	15	Strategies for achieving adequate participant enrolment to reach target sample size	4,8
**Methods: Assignment of interventions (for controlled trials)**			
Allocation:			
Sequence generation	16a	Method of generating the allocation sequence (eg, computer-generated random numbers), and list of any factors for stratification. To reduce predictability of a random sequence, details of any planned restriction (eg, blocking) should be provided in a separate document that is unavailable to those who enrol participants or assign interventions	7
Allocation concealment mechanism	16b	Mechanism of implementing the allocation sequence (eg, central telephone; sequentially numbered, opaque, sealed envelopes), describing any steps to conceal the sequence until interventions are assigned	7
Implementation	16c	Who will generate the allocation sequence, who will enrol participants, and who will assign participants to interventions	7
Blinding (masking)	17a	Who will be blinded after assignment to interventions (eg, trial participants, care providers, outcome assessors, data analysts), and how	7
	17b	If blinded, circumstances under which unblinding is permissible, and procedure for revealing a participant's allocated intervention during the trial	NA
**Methods: Data collection, management, and analysis**			

Data collection methods	18a	Plans for assessment and collection of outcome, baseline, and other trial data, including any related processes to promote data quality (eg, duplicate measurements, training of assessors) and a description of study instruments (eg, questionnaires, laboratory tests) along with their reliability and validity, if known. Reference to where data collection forms can be found, if not in the protocol	6
	18b	Plans to promote participant retention and complete follow-up, including list of any outcome data to be collected for participants who discontinue or deviate from intervention protocols	9
Data management	19	Plans for data entry, coding, security, and storage, including any related processes to promote data quality (eg, double data entry; range checks for data values). Reference to where details of data management procedures can be found, if not in the protocol	NA
Statistical methods	20a	Statistical methods for analysing primary and secondary outcomes. Reference to where other details of the statistical analysis plan can be found, if not in the protocol	7-8
	20b	Methods for any additional analyses (eg, subgroup and adjusted analyses)	7-8
	20c	Definition of analysis population relating to protocol non-adherence (eg, as randomised analysis), and any statistical methods to handle missing data (eg, multiple imputation)	7-8
**Methods: Monitoring**			
Data monitoring	21a	Composition of data monitoring committee (DMC); summary of its role and reporting structure; statement of whether it is independent from the sponsor and competing interests; and reference to where further details about its charter can be found, if not in the protocol. Alternatively, an explanation of why a DMC is not needed	NA
	21b	Description of any interim analyses and stopping guidelines, including who will have access to these interim results and make the final decision to terminate the trial	8

For peer review only - http://bmjopen.bmj.com/site/about/guidelines.xhtml

Harms	22	Plans for collecting, assessing, reporting, and managing solicited and spontaneously reported adverse events and other unintended effects of trial interventions or trial conduct	NA
Auditing	23	Frequency and procedures for auditing trial conduct, if any, and whether the process will be independent from investigators and the sponsor	NA
**Ethics and dissemination**			
Research ethics approval	24	Plans for seeking research ethics committee/institutional review board (REC/IRB) approval	9
Protocol amendments	25	Plans for communicating important protocol modifications (eg, changes to eligibility criteria, outcomes, analyses) to relevant parties (eg, investigators, REC/IRBs, trial participants, trial registries, journals, regulators)	NA
Consent or assent	26a	Who will obtain informed consent or assent from potential trial participants or authorised surrogates, and how (see Item 32)	4-5
	26b	Additional consent provisions for collection and use of participant data and biological specimens in ancillary studies, if applicable	NA
Confidentiality	27	How personal information about potential and enrolled participants will be collected, shared, and maintained in order to protect confidentiality before, during, and after the trial	NA
Declaration of interests	28	Financial and other competing interests for principal investigators for the overall trial and each study site	12
Access to data	29	Statement of who will have access to the final trial dataset, and disclosure of contractual agreements that limit such access for investigators	9
Ancillary and post-trial care	30	Provisions, if any, for ancillary and post-trial care, and for compensation to those who suffer harm from trial participation	NA
Dissemination policy	31a	Plans for investigators and sponsor to communicate trial results to participants, healthcare professionals, the public, and other relevant groups (eg, via publication, reporting in results databases, or other data sharing arrangements), including any publication restrictions	9

For peer review only

	31b	Authorship eligibility guidelines and any intended use of professional writers	NA
	31c	Plans, if any, for granting public access to the full protocol, participant-level dataset, and statistical code	9
**Appendices**			
Informed consent materials	32	Model consent form and other related documentation given to participants and authorised surrogates	Appendix A
Biological specimens	33	Plans for collection, laboratory evaluation, and storage of biological specimens for genetic or molecular analysis in the current trial and for future use in ancillary studies, if applicable	NA

*It is strongly recommended that this checklist be read in conjunction with the SPIRIT 2013 Explanation & Elaboration for important clarification on the items. Amendments to the protocol should be tracked and dated. The SPIRIT checklist is copyrighted by the SPIRIT Group under the Creative Commons "Attribution-NonCommercial-NoDerivs 3.0 Unported" license.

For peer review only

BMJ Open

Effects of a waiting list control design on alcohol consumption among online help-seekers: protocol for a randomised controlled trial

Journal:	*BMJ Open*
Manuscript ID	bmjopen-2021-049810.R2
Article Type:	Protocol
Date Submitted by the Author:	11-Aug-2021
Complete List of Authors:	Bendtsen, Marcus; Linköping University<br>Ulfsdotter Gunnarsson, Katarina; Linköping University<br>McCambridge, Jim; University of York, Department of Health Sciences
**Primary Subject Heading**:	Public health
Secondary Subject Heading:	Epidemiology, Ethics
Keywords:	STATISTICS & RESEARCH METHODS, PUBLIC HEALTH, PREVENTIVE MEDICINE

SCHOLARONE™
Manuscripts

I, the Submitting Author has the right to grant and does grant on behalf of all authors of the Work (as defined in the below author licence), an exclusive licence and/or a non-exclusive licence for contributions from authors who are: i) UK Crown employees; ii) where BMJ has agreed a CC-BY licence shall apply, and/or iii) in accordance with the terms applicable for US Federal Government officers or employees acting as part of their official duties; on a worldwide, perpetual, irrevocable, royalty-free basis to BMJ Publishing Group Ltd ("BMJ") its licensees and where the relevant Journal is co-owned by BMJ to the co-owners of the Journal, to publish the Work in this journal and any other BMJ products and to exploit all rights, as set out in our licence.

The Submitting Author accepts and understands that any supply made under these terms is made by BMJ to the Submitting Author unless you are acting as an employee on behalf of your employer or a postgraduate student of an affiliated institution which is paying any applicable article publishing charge ("APC") for Open Access articles. Where the Submitting Author wishes to make the Work available on an Open Access basis (and intends to pay the relevant APC), the terms of reuse of such Open Access shall be governed by a Creative Commons licence – details of these licences and which Creative Commons licence will apply to this Work are set out in our licence referred to above.

Other than as permitted in any relevant BMJ Author's Self Archiving Policies, I confirm this Work has not been accepted for publication elsewhere, is not being considered for publication elsewhere and does not duplicate material already published. I confirm all authors consent to publication of this Work and authorise the granting of this licence.

For peer review only

EFFECTS OF A WAITING LIST CONTROL DESIGN ON ALCOHOL CONSUMPTION AMONG ONLINE HELP-SEEKERS: PROTOCOL FOR A RANDOMISED CONTROLLED TRIAL

Marcus Bendtsen[1,*], Katarina Ulfsdotter Gunnarsson[1], Jim McCambridge[2]

[1] Department of Health, Medicine and Caring Sciences, Linköping University, Sweden.

[2] Department of Health Sciences, University of York.

*Corresponding author

Marcus Bendtsen (marcus.bendtsen@liu.se)

Department of Health, Medicine and Caring Sciences, Division of Society and Health

Linköping University, 581 83 Linköping, registrator@liu.se, +46 13 28 10 00

Word count: 3369

For peer review only

ABSTRACT

Introduction: Sparse attention has been given to the design of control conditions in trials, despite their important role as contrasts for novel treatments, and thus as a key determinant of effect sizes. This undermines valid inferences on effect estimates in trials, which are fundamentally comparative in nature. Such challenges to understanding also makes generalisation of effect estimates complex, for example it may not be clear to what degree real-world alternatives to the novel treatments in pragmatic trials are similar to the control conditions studied. The present study aims to estimate the effects of being allocated to a waiting list control condition.

Methods and analysis: Individuals searching online for help to reduce their drinking will be invited to take part in a study. Individuals aged 18 or older, who in the past month consumed 6 or more drinks on one occasion, or consumed 10 or more drinks the past week, will be eligible to participate. Both groups will receive identical feedback and advice on behaviour change, however, one group will be informed that they have to wait one month for the intervention materials. One month post-randomisation, participants will receive an email with the follow-up questionnaire measuring the primary outcomes: (1) frequency of heavy episodic drinking (defined as at study entry) in the past month; and (2) overall past week alcohol consumption. Differences between groups will be analysed using negative binomial regression models estimated using Bayesian inference. Recruitment will begin in October 2021. A Bayesian group sequential design will be employed to determine when to end enrolment (expected to be between 500 and 1500 individuals).

Ethics and dissemination: The study was approved by the Swedish Ethical Review Authority on 2021-01-25 (Dnr 2020-06267). Findings will be disseminated in open access peer-reviewed journals no later than 2023.

Registration: Prospectively registered on 2021-02-01 (ISRCTN14959594).

ARTICLE SUMMARY

STRENGTHS AND LIMITATIONS OF THIS STUDY

- Sparse attention has been given to the effects of different control conditions.
- Use of a double blind randomised controlled design allows for a valid estimate of the effects of being allocated to a waiting list control.
- The Bayesian group sequential design will allow for recruitment to not last longer than necessary, minimizing the number of participants exposed to the necessary deception involved in the study.
- The trial is an online trial, which usually suffer from high rates of attrition.

INTRODUCTION

Effects estimated in randomised controlled trials (RCTs) should always be understood as contrasts between allocated conditions [1]. Since effects are contrasts, estimates are affected by either side of the comparison. While much effort is usually placed in designing and describing the novel treatment side of the contrast, typically a fraction of this attention is given to the control side. There is sparse attention to the design of control conditions in trials in the research literature [2–6]. Not only does this potentially introduce bias, and certainly complicates the interpretation of the effects estimated, it also makes generalisability problematic, with implications for research use in decision-making.

To illustrate the complex nature of the generalisability issues, we borrow notation from the potential outcomes framework [7]. Let Y_x represent the outcome for an individual if the individual would hypothetically receive treatment x. There may be a range of different treatments available, and depending on which treatment an individual actually receives, different outcomes will follow. In a 2-arm RCT, the two *potential* outcomes for participants are Y_0 and Y_1 , i.e., the outcomes realised if they are allocated to the control condition ($x = 0$) or the intervention condition ($x = 1$). The individual level causal effect of the intervention is defined as $Y_1 - Y_0$, that is, the potential outcome under the intervention condition minus the potential outcome under the control condition (or some other mode of contrast, e.g. risk or odds). Of course, when using between-subjects designs it will be impossible to know both Y_1 and Y_0 for the same individual, thus RCTs are generally focused on the population level causal effect. With continuous outcomes, this is commonly the expected difference $E[Y_1] - E[Y_0]$ between the two conditions. We rely on the license given to us by virtue of randomisation to claim that these are unconfounded estimates with sufficiently large numbers, protecting the internal validity of our estimates.

Ideally, $E[Y_1] - E[Y_0]$ would be an estimate which arises from an effectiveness or pragmatic trial rather than an efficacy or explanatory trial [8,9], which can be interpreted as the effect anticipated in a real-world roll-out. Y_1 could be a novel potential outcome which we may offer to individuals, and it may be close to what was estimated in the trial if the study inclusion criteria were similar to routine practice, there were no marked bias, and if treatment protocols are followed. However, a problem arises if Y_0 is not the potential outcome which would be the alternative to Y_1 in the real world. For instance, if Y_0 represents the potential outcome of being placed on a waiting list [10], then that is not an outcome which is available to individuals in a real-world setting, except in those situations where there are waiting lists for treatment. To avoid any such problem the control condition in the trial needs to be identical to usual care. If being placed on a waiting list in a study affects individuals in any way differently than do the real-world alternatives to Y_1, then the external validity of the contrast $E[Y_1] - E[Y_0]$ should be questioned, and measures need to be taken to facilitate the interpretation of the observed effect.

WAITING LIST CONTROLS

Rather than withholding treatment from controls in RCTs altogether, it has been argued that it is more ethical to offer the treatment to controls after the trial period [10] – i.e. placing participants on a waiting list. This type of control condition is commonly used in behavioural and rehabilitation treatments [11], altering the nature of Y_0. It is not well understood how individuals react to these decisions. For example, in one smoking cessation trial some control participants had negative feelings about having to wait, and decided to quit later [12]. Researchers have long been concerned with feelings of disappointment about allocation to control conditions in trials more broadly, but we know little about how study participants subsequently act on them [13,14]. This may be especially the case in online studies when participants actively engage with recruitment procedures and may therefore have higher expectations of receiving novel treatment, rather than simply respond to in-person contacts from researchers that they have not initiated. On the other hand, the effects of being

For peer review only - http://bmjopen.bmj.com/site/about/guidelines.xhtml

informed that one has to wait, as investigated here, may be less pronounced in online studies [15–19]. It therefore remains unclear if these reactions translate into any markedly biased effect estimates.

Indirect evidence suggests that in many circumstances we may expect participants in control conditions to change more than they ordinarily would if not involved in a study. It has for instance been found that alcohol consumption tends to decrease in control groups by approximately 20% in alcohol intervention trials [20–22]. This change is likely due to a combination of several factors, including regression to the mean [23], assessment effects [24], and other forms of research participation effects [25–27]. A network meta-analysis of cognitive behaviour therapy for depression [28], found in contrast that participants in waiting list control groups improved less than expected. This is in line with thinking that when patients are placed on waiting lists, they are to some extent compliant with the implicit direction; they wait, and avoid instigating changes [29].

A direct estimation of the effects of a waiting list control design on alcohol consumption was conducted among 185 hazardous or harmful drinkers recruited through newspaper advertisements in Canada [30]. A 2-arm parallel groups RCT was employed, in which both groups received the same brief alcohol intervention, but one group were told that they had been allocated to the intervention arm, and the other told they were allocated to a waiting list arm. This exploratory trial found an interaction between being allocated to the waiting list group and the action subscale of the readiness to change questionnaire [31,32]. This suggested that those in the waiting list group who rated themselves above the median on the action subscale (thus being more ready for action) reported consuming more alcohol in comparison to their counterparts in the intervention group (approximately 6 drinks more per week and 1.3 more drinks on the occasion when they drank the most). They thus waited to reduce their drinking. While findings from the trial were mixed, it generated hypotheses important to study further. The current trial described in this protocol will address these hypotheses with a larger sample size in an online recruitment setting.

OBJECTIVES

Using a 2-arm (1:1) randomised controlled trial, the objective of this study is to estimate the effects of being allocated to a waiting list control group on alcohol consumption. The primary effects being estimated are the total effect and moderation by readiness to change. We anticipate that these effects will be in the direction of higher consumption at follow-up among waiting list participants in comparison to non-waiting list participants.

METHODS AND ANALYSIS

This protocol contains relevant items from the Standard Protocol Items: Recommendations for Interventional Trials (SPIRIT) [33]. The trial has been prospectively registered in the ISRCTN registry on 2021-02-01 (ISRCTN14959594), and has received ethical approval by the Swedish Ethical Review Authority on 2021-01-25 (Dnr 2020-06267).

STUDY SETTING, ELIGIBILITY CRITERIA AND RECRUITMENT

Participants will be recruited through online advertisements (Google Ads), using language targeting to include individuals using Google's services in English (including search and gmail). Keywords will be selected to target those seeking help to drink less, e.g. "How do I drink less", "I drink too much", and "Support for drinkers". The adverts will be designed to target individuals who are willing to take part in a trial of a self-help alcohol intervention. An example advert is shown in Figure 1, and study information presented to individuals who click on the advert can be found in Appendix A. Recruitment will begin in October 2021 and last for no more than 24 months (October 2023, see Sample Size).

INSERT FIGURE1 HERE

For peer review only - http://bmjopen.bmj.com/site/about/guidelines.xhtml

Figure 1 - Example advert shown to individuals who search online for help to drink less

Individuals who consent to take part in the trial will be asked to complete a baseline questionnaire (see Appendix B). Individuals will first be asked to state their age and sex, the number of times during the past month they consumed 6 or more drinks on one occasion, and the number of drinks consumed in the past week. Individuals will be included if they report having consumed 6 or more drinks on one occasion at least once in the past month or having consumed more than 9 drinks in the past week. Individuals younger than 18 years old will be excluded. Those not excluded will be asked to complete the readiness to change questionnaire. After completing the questionnaire, individuals will be asked to leave an email address to which further information about study participation will be sent. The email will be sent immediately and will include a brief text explaining that a link should be clicked in the email to continue participation in the study. Individuals who click on the link in the email will be randomised to either the intervention group (IG) or the waiting list control group (WL).

INTERVENTIONS

Participants in both groups (IG and WL) will receive the same personalised feedback and advice based on the baseline questionnaire. The material, which has been developed from our previous research [24,34–40] and is based around social cognitive theories of health behaviour [41], includes: (1) feedback on current consumption in relation to recommendations, (2) content to increase motivation and self-efficacy, and (3) advice on what to do to reduce drinking and avoid environmental triggers.

Preceding the feedback and advice will be a page with study information which will be different between groups; this is the experimental contrast in the present study.

Text to IG group
There are two groups in this study. You are in the group which receives immediate feedback and support tailored to the responses to the questionnaire you previously completed. This material has been designed for people who are concerned about their drinking. [Button: Take me to the feedback].

Text to WL group
There are two groups in this study. You are in the group which will have to wait for the feedback and support material. We will contact you again in four weeks, and at that time you will be given the material. In the meantime, we have generated a report based on your responses to the questionnaire previously completed. [Button: Take me to the feedback].

OUTCOMES

Primary:

1. Frequency of heavy episodic drinking in the past month
2. Overall consumption the past week

Secondary:

3. Readiness to change

The two primary outcomes have been selected to be similar to the outcomes used in the exploratory trial upon which this trial has been modelled [30], while at the same time being part of the proposed core outcome set for brief alcohol interventions [42,43]. Frequency of heavy episodic drinking will be defined as the number of times in the past month the participant has consumed 6 or more drinks on one occasion. Number of drinks consumed in the past week will be assessed by asking participants to report their consumption day-by-day.

For peer review only - http://bmjopen.bmj.com/site/about/guidelines.xhtml

The secondary outcome, readiness to change, will be measured using the readiness to change questionnaire (treatment version) [31,32]. While our primary use of this scale is for assessing moderation effects (using the baseline assessment), we are also interested in estimating effects of the WL condition on readiness to change directly.

FOLLOW-UP

One month post randomisation, participants will be sent an email including a link to the follow-up questionnaire (see Appendix B). Participants will be sent two reminders three days apart. If no response has been recorded three days after the final reminder, a fourth email will be sent with the frequency of heavy episodic drinking in the past month measure embedded in the email. Reminders will only be sent to participants who have not yet responded to the two primary alcohol consumption outcomes. Participants in the WL group will immediately after completing the outcome measure be sent an email with information about the deception involved in the study (Appendix C). A participant timeline is shown in Figure 2.

INSERT FIGURE2 HERE

Figure 2 - Participant timeline (SPIRIT figure)

ALLOCATION AND BLINDING

Block randomisation will be used with randomly permuted block sizes of 2 and 4 to ensure equal size groups. Randomisation will be automatic and computerised on the backend server, thus neither research personnel nor participants will be able to influence allocation. Participants will be blind to the true nature of the study. Research personnel will be blind to allocation.

STATISTICAL METHODS

All analyses will be conducted according to intention-to-treat principles, with all participants analysed in the groups to which they were randomised. Analyses will be done using available data with missing data imputed. Attrition analyses will explore the plausibility of the implicit missing at random (MAR) assumption underlying the analyses. Model parameters will be interpreted by inspecting marginal posterior distributions using Bayesian inference (see Sample Size for priors) [44], and imputation will be done using multiple imputation with chained equations. We will use R version 3.6 with the RStan library for all analyses [45].

PRIMARY ANALYSES

The analysis of primary and secondary outcomes will be conducted through regression models in which each outcome will be regressed against group allocation. One regression model per outcome will be created. Negative binomial regression will be used for both primary outcomes and linear regression will be used for readiness to change scores (precontemplation, contemplation and action). A multinomial regression model will also be estimated for stage of change designation (i.e. highest arithmetical score among the three sub scales with ties preferring the stage farther along the continuum of change [31,32]). All regression models will be adjusted for age, sex and each respective outcome measured at baseline.

EXPLORATORY ANALYSES

Effect modification analyses of group allocation and readiness to change scores (precontemplation, contemplation and action) on the primary outcomes will be analysed by adding interaction terms to the respective regression models. We will estimate separate models for each stage interaction, and a combined

For peer review only - http://bmjopen.bmj.com/site/about/guidelines.xhtml

model with all stages interacting with allocation using shrinkage priors [46]. We will also explore effect modification with respect to group allocation and stage of change designation on the primary outcomes.

Separate analyses of the heavy episodic drinking outcome (first primary outcome) will be conducted among those who at baseline had consumed 6 or more drinks on one or more occasions over the past month – thus excluding those who had no episodes of heavy drinking the month preceding enrolment. Similarly, separate analyses of the overall consumption the past week (second primary outcome) will be conducted among those who at baseline reported being at-risk with respect to weekly consumption – thus excluding those who at baseline reported having consumed 9 drinks or fewer the past week.

MEDIATOR ANALYSES

We will estimate the degree to which the effect of allocation on the primary outcomes is mediated through two variables: (1) accessing the intervention materials and (2) time spent on the intervention materials. We will use a causal inference framework [47–49], using Bayesian inference to estimate the natural direct effect and natural indirect effect (as per the definitions of Pearl [49]). We will report on the posterior distributions of these two estimates, as well as the proportion of the total effect which is accounted for by the natural indirect effect. We will also explore if readiness to change moderates any of these effects.

ATTRITION ANALYSES

The available data and imputation approach used in the primary analyses may result in biased intention-to-treat estimates if data is not MAR. We will therefore seek evidence against the MAR assumption in two attrition analyses. First, since late responders to follow-up may be more alike non-responders than early responders are, we will investigate if outcomes are systematically different given the number of reminders needed to collect follow-up data. Second, we will explore if responders and non-responders are systematically different with respect to baseline characteristics, using logistic regression models with shrinkage priors.

SAMPLE SIZE

The trial will use a Bayesian group sequential design to monitor recruitment with interim analyses planned for every 25 participants completing the follow-up questionnaire [50–52]. The primary outcomes will be modelled following the analysis plan presented earlier, and each covariate representing group allocation will be assessed for evidence of effect or futility. Covariates representing the interaction between the stages of readiness for change and group allocation will also be monitored (using separate models for each stage interaction). Let β_i represent the covariates of interest, and D the data available, then for each β_i the target criteria will be:

- **Effect**: $p(\beta_i > 0|D) > 97.5\%$ or $p(\beta_i < 0|D) > 97.5\%$ (i.e. the effect is greater (or less) than 0 with a probability greater than 97.5%).

- **Futility**: $p(\log(\frac{1}{1.10}) < \beta_i < \log(1.10)|D) > 95.0\%$ (i.e. the incidence rate ratio is greater than 1/1.10 and less than 1.10 with a probability greater than 95%).

For the effect criterion, we will use a sceptical normal prior for covariates (mean = 0 and standard deviation = 1.0), and a wider prior will be used for the futility criterion (mean = 0 and standard deviation = 2.0).

The criteria above should be viewed as targets, thus at each interim analysis we will evaluate each criterion for each covariate and make a decision if we believe that recruitment should end. Simulations indicate that we will require a sample size in the range of 500 to 1500 participants. Recruitment will not exceed 24 months.

For peer review only - http://bmjopen.bmj.com/site/about/guidelines.xhtml

PATIENT AND PUBLIC INVOLVEMENT STATEMENT

Patients were not involved in the planning of this study.

DISCUSSION

Trials are usually advertised as important for research, but they may also be seen as an opportunity for prospective participants to get access to novel treatments. Participants have likely found the trial, and enrolled, motivated by the ways in which the trial may benefit them. However, trials do not primarily advertise the conditions which lead to Y_0 , i.e., recruitment material never says “Do you want to be on a waiting list?”. The unintended consequences of telling participants that they will have to wait is the motivation for the present study, which is designed to uncover some of these consequences in the chosen setting.

The findings from the present study will help to understand if participants are affected by being told that they are being placed in a waiting list control group and will therefore help to guide decisions of comparator choice in future studies of brief alcohol interventions. In addition, effect estimates from the study will also support the possibility of retrospectively adjusting findings from previous trials of brief alcohol interventions to account for any bias induced by waiting list controls [53].

LIMITATIONS

Online studies of alcohol interventions commonly suffer from high levels of attrition, particularly at follow-up, and we expect the primary limitation of this study to be high attrition rates. To reduce attrition, we will only randomise participants after they have confirmed their email address, which helps to ensure that we have a valid email address for each randomised participant. We will use reminders over a short time span to keep participants aware that it is important that they respond, and finally make it easy for them to respond by including one of the primary outcome measures in the body of the email. The Bayesian group sequential design will ensure that we collect data for as long as is necessary to fulfil the criteria (see Sample Size), which will ensure that we have enough samples for our analyses. However, this will not protect against differential attrition, and thus does not protect against attrition bias, so we will need to study this risk carefully.

ETHICS AND DISSEMINATION

The main ethical concern in this trial is the use of deception - required to not reveal to prospective participants that it is the effects of allocation which are being studied. Routine use of deception in research is not recommended, however, considering the widespread use of waiting list designs, understanding their effects was considered important and justified the risks induced by deception. The study was approved by the Swedish Ethical Review Authority on 2021-01-25 (Dnr 2020-06267). Data collection will begin in October 2021 and last no longer than 24 months. The dataset will be made available to researchers upon reasonable request. Findings will be published in peer-reviewed journals and presented at relevant conferences no later than 2023.

REFERENCES

1. Barkauskas VH, Lusk SL, Eakin BL. Selecting Control Interventions for Clinical Outcome Studies. West J Nurs Res. 2005 Apr;27(3):346–63.

2. Cook TD, Campbell DT. Quasi-Experimentation: Design and Analysis Issues for Field Settings. Houghton Mifflin; 1979.

For peer review only - http://bmjopen.bmj.com/site/about/guidelines.xhtml

3. Rosnow RL, Rosenthal R. People studying people: Artifacts and ethics in behavioral research. New York: W.H. Freeman; 1997.

4. Freedland KE, King AC, Ambrosius WT, Mayo-Wilson E, Mohr DC, Czajkowski SM, et al. The selection of comparators for randomized controlled trials of health-related behavioral interventions: recommendations of an NIH expert panel. J Clin Epidemiol. 2019 Jun;110:74–81.

5. Freedland KE. Demanding Attention: reconsidering the role of attention control groups in behavioral intervention research. Psychosom Med. 2013;75(2):100–2.

6. Freedland KE, Mohr DC, Davidson KW, Schwartz JE. Usual and Unusual Care: existing practice control groups in randomized controlled trials of behavioral interventions. Psychosom Med. 2011 May;73(4):323–35.

7. Rubin DB. Estimating causal effects of treatments in randomized and nonrandomized studies. J Educ Psychol. 1974;66(5):688–701.

8. Singal AG, Higgins PDR, Waljee AK. A Primer on Effectiveness and Efficacy Trials. Clin Transl Gastroenterol. 2014 Jan;5(1):e45.

9. Roland M, Torgerson DJ. Understanding controlled trials: What are pragmatic trials? BMJ. 1998 Jan 24;316(7127):285–285.

10. Elliott SA, Brown JSL. What are we doing to waiting list controls? Behav Res Ther. 2002 Sep;40(9):1047–52.

11. Hart T, Bagiella E. Design and Implementation of Clinical Trials in Rehabilitation Research. Arch Phys Med Rehabil. 2012 Aug;93(8):S117–26.

12. Müssener U, Linderoth C, Bendtsen M. Exploring the Experiences of Individuals Allocated to a Control Setting: Findings From a Mobile Health Smoking Cessation Trial. JMIR Hum Factors. 2019;6(2):e12139.

13. McCambridge J, Sorhaindo A, Quirk A, Nanchahal K. Patient preferences and performance bias in a weight loss trial with a usual care arm. Patient Educ Couns. 2014 May;95(2):243–7.

14. Howick J. Questioning the Methodologic Superiority of 'Placebo' Over 'Active' Controlled Trials. Am J Bioeth. 2009 Sep 11;9(9):34–48.

15. McCambridge J, Wilson A, Attia J, Weaver N, Kypri K. Randomized trial seeking to induce the Hawthorne effect found no evidence for any effect on self-reported alcohol consumption online. J Clin Epidemiol. 2019 Apr;108:102–9.

16. Kypri K, Wilson A, Attia J, Sheeran P, Miller P, McCambridge J. Social Desirability Bias in the Reporting of Alcohol Consumption: A Randomized Trial. J Stud Alcohol Drugs. 2016 May;77(3):526–31.

17. McCambridge J. From question-behaviour effects in trials to the social psychology of research participation. Psychol Health. 2015 Jan 2;30(1):72–84.

18. Kypri K, Wilson A, Attia J, Sheeran PJ, McCambridge J. Effects of study design and allocation on self-reported alcohol consumption: randomized trial. Trials. 2015 Dec;16(1):127.

19. Kypri K, Bowe SJ, Karlsson N, McCambridge J. Enrolment-latency in randomized behavior change trials: individual participant data meta-analysis showed association with attrition but not effect-size. J Clin Epidemiol. 2020 Feb;118:55–9.

For peer review only

20. Jenkins RJ, McAlaney J, McCambridge J. Change over time in alcohol consumption in control groups in brief intervention studies: systematic review and meta-regression study. Drug Alcohol Depend. 2009 Feb;100(1–2):107–14.

21. Jenkins RJ, McAlaney J, McCambridge J. Corrigendum to "Change over time in alcohol consumption in control groups in brief intervention studies: Systematic review and meta-regression study" [Drug Alcohol Depend. 100 (2009) 107–114]. Drug Alcohol Depend. 2010 Apr;108(1–2):151.

22. Moyer A, Finney JW. Outcomes for untreated individuals involved in randomized trials of alcohol treatment. J Subst Abuse Treat. 2002 Oct;23(3):247–52.

23. Linden A. Assessing regression to the mean effects in health care initiatives. BMC Med Res Methodol. 2013 Dec 28;13(1):119.

24. McCambridge J, Bendtsen M, Karlsson N, White IR, Nilsen P, Bendtsen P. Alcohol assessment and feedback by email for university students: Main findings from a randomised controlled trial. Br J Psychiatry. 2013;203(5):334–40.

25. McCambridge J, Witton J, Elbourne DR. Systematic review of the Hawthorne effect: New concepts are needed to study research participation effects. J Clin Epidemiol. 2014 Mar;67(3):267–77.

26. McCambridge J, Kypri K, Elbourne D. Research participation effects: a skeleton in the methodological cupboard. J Clin Epidemiol. 2014 Aug;67(8):845–9.

27. McCambridge J, Kypri K, McElduff P. Regression to the mean and alcohol consumption: A cohort study exploring implications for the interpretation of change in control groups in brief intervention trials. Drug Alcohol Depend. 2014 Feb;135:156–9.

28. Furukawa TA, Noma H, Caldwell DM, Honyashiki M, Shinohara K, Imai H, et al. Waiting list may be a nocebo condition in psychotherapy trials: a contribution from network meta-analysis. Acta Psychiatr Scand. 2014 Sep;130(3):181–92.

29. Miller W, Rollnick S. Motivational Interviewing: Preparing People for Change. New York: Guilford Press; 2002.

30. Cunningham JA, Kypri K, McCambridge J. Exploratory randomized controlled trial evaluating the impact of a waiting list control design. BMC Med Res Methodol. 2013 Dec;13(1):150.

31. Rollnick S, Heather N, Gold R, Hall W. Development of a short 'readiness to change' questionnaire for use in brief, opportunistic interventions among excessive drinkers. Addiction. 1992 May;87(5):743–54.

32. Heather N, Hönekopp J. A revised edition of the Readiness to Change Questionnaire [Treatment Version]. Addict Res Theory. 2008 Jan 11;16(5):421–33.

33. Chan A-W, Tetzlaff JM, Altman DG, Laupacis A, Gøtzsche PC, Krleža-Jerić K, et al. SPIRIT 2013 Statement: Defining Standard Protocol Items for Clinical Trials. Ann Intern Med. 2013 Feb 5;158(3):200.

34. Bendtsen P, McCambridge J, Bendtsen M, Karlsson N, Nilsen P. Effectiveness of a Proactive Mail-Based Alcohol Internet Intervention for University Students: Dismantling the Assessment and Feedback Components in a Randomized Controlled Trial. J Med Internet Res. 2012 Oct 31;14(5):e142.

35. McCambridge J, Bendtsen P, Bendtsen M, Nilsen P. Alcohol email assessment and feedback study dismantling effectiveness for university students (AMADEUS-1): study protocol for a randomized controlled trial. Trials. 2012 Dec 6;13(1):49.

For peer review only

36. McCambridge J, Bendtsen M, Karlsson N, White IR, Bendtsen P. Alcohol assessment & feedback by e-mail for university student hazardous and harmful drinkers: Study protocol for the AMADEUS-2 randomised controlled trial. BMC Public Health. 2013;13(1):949.

37. Bendtsen P, Bendtsen M, Karlsson N, White IR, McCambridge J. Online Alcohol Assessment and Feedback for Hazardous and Harmful Drinkers: Findings From the AMADEUS-2 Randomized Controlled Trial of Routine Practice in Swedish Universities. J Med Internet Res. 2015;17(7):e170.

38. Bendtsen M. An Electronic Screening and Brief Intervention for Hazardous and Harmful Drinking Among Swedish University Students: Reanalysis of Findings From a Randomized Controlled Trial Using a Bayesian Framework. J Med Internet Res. 2019;21(12):e14420.

39. Bendtsen M. Electronic Screening for Alcohol Use and Brief Intervention by Email for University Students: Reanalysis of Findings From a Randomized Controlled Trial Using a Bayesian Framework. J Med Internet Res. 2019;21(11):e14419.

40. Bendtsen M, McCambridge J. Reducing Alcohol Consumption Among Risky Drinkers in the General Population of Sweden Using an Interactive Mobile Health Intervention: Protocol for a Randomized Controlled Trial. JMIR Res Protoc. 2019;8(4):e13119.

41. Conner M, Norman P. Predicting Health Behavior: Research and Practice with Social Cognition Models. 2005.

42. Shorter GW, Bray JW, Giles EL, O'Donnell AJ, Berman AH, Holloway A, et al. The Variability of Outcomes Used in Efficacy and Effectiveness Trials of Alcohol Brief Interventions: A Systematic Review. J Stud Alcohol Drugs. 2019 May;80(3):286–98.

43. Shorter GW, Heather N, Bray JW, Berman AH, Giles EL, O'Donnell AJ, et al. Prioritization of Outcomes in Efficacy and Effectiveness of Alcohol Brief Intervention Trials: International Multi-Stakeholder e-Delphi Consensus Study to Inform a Core Outcome Set. J Stud Alcohol Drugs. 2019 May;80(3):299–309.

44. Bendtsen M. A Gentle Introduction to the Comparison Between Null Hypothesis Testing and Bayesian Analysis: Reanalysis of Two Randomized Controlled Trials. J Med Internet Res. 2018;20(10):e10873.

45. Team SD. RStan: the R interface to Stan. 2020.

46. Piironen J, Vehtari A. Sparsity information and regularization in the horseshoe and other shrinkage priors. Electron J Stat. 2017;11(2):5018–51.

47. Imai K, Keele L, Tingley D. A General Approach to Causal Mediation Analysis. Psychol Methods. 2010;15(4):309–34.

48. Pearl J. Causality. Causality: Models, Reasoning, and Inference, Second Edition. Cambridge: Cambridge University Press; 2009.

49. Pearl J. Interpretation and identification of causal mediation. Psychol Methods. 2014;19(4):459–81.

50. Gsponer T, Gerber F, Bornkamp B, Ohlssen D, Vandemeulebroecke M, Schmidli H. A practical guide to Bayesian group sequential designs. Pharm Stat. 2014;13(1):71–80.

51. Berry DA. Bayesian clinical trials. Nat Rev Drug Discov. 2006 Jan;5(1):27–36.

52. Bendtsen M. The P Value Line Dance: When Does the Music Stop? J Med Internet Res. 2020;22(8):e21345.

53. Bendtsen M, McCambridge J. Causal models accounted for research participation effects when estimating effects in a behavioral intervention trial. J Clin Epidemiol. 2021 Mar 13;136:77–83.

For peer review only

AUTHORS' CONTRIBUTIONS

MB and JM defined the study objectives and designed the trial. All authors were involved in developing the intervention materials. MB and KUG are responsible for data collection and statistical analysis. All authors are involved in interpreting findings and all authors read and approved the protocol manuscript.

FUNDING

This research received no specific grant from any funding agency in the public, commercial or not-for-profit sectors.

COMPETING INTERESTS

MB owns a private company (Alexit AB) which disseminates digital lifestyle interventions such as the one offered to participants in this trial. Alexit AB had no part in funding, planning, or execution of this study.

DATA AVAILABILITY STATEMENT

Deidentified individual participant-level data will be made available upon reasonable request following publication. The data may only be used for analyses that are congruent with the informed consent given by participants and the ethical approval of the trial. A registered study protocol must be available before data is shared.

FIGURE LEGENDS

Figure 3 - Example advert shown to individuals who search online for help to drink less

Figure 2 - Participant timeline (SPIRIT figure)

For peer review only

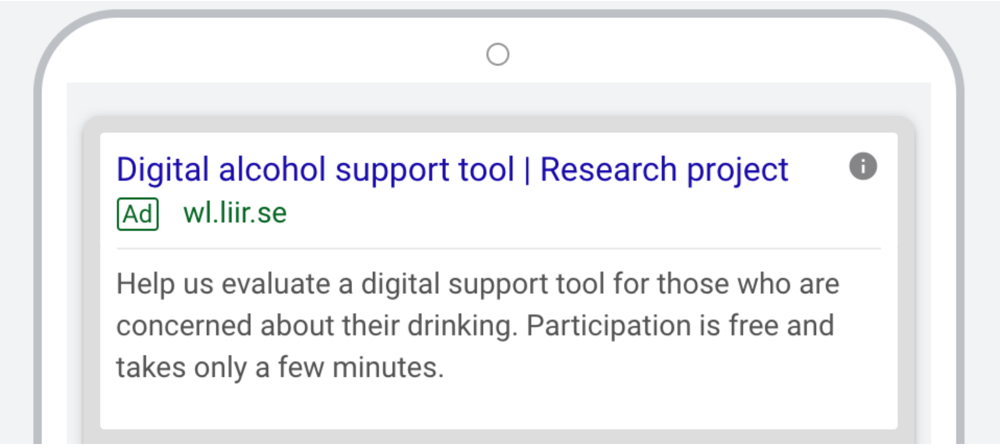

Example advert shown to individuals who search online for help to drink less

	STUDY PERIOD			
	**Enrolment**	**Allocation**	**Post-allocation**	**Close-out**
**TIMEPOINT**	**0**	**0**	***Access to feedback and support material***	***1-month***
**ENROLMENT:**				
Informed consent	X			
Eligibility screen	X			
Allocation		X		
Debriefing				X
**INTERVENTIONS:**				
Intervention group (IG)			↔	
Waiting list group (WL)			↔	
**ASSESSMENTS:**				
Baseline questionnaire	X			
Follow-up questionnaire				X
Accessing material			X	X
Time spent on material			X	

For peer review only

For peer review only - http://bmjopen.bmj.com/site/about/guidelines.xhtml

APPENDIX A – INFORMED CONSENT MATERIALS

When clicking on the online advert, participants will be shown the following information about the study.

Do you want to participate in the "Digital support for alcohol" study?

Thank you for showing interest in this study. By pressing the button "I consent to take part in this study" you are consenting to take part in the study described below.

Why are we doing this study and why do you want me to participate? We have developed a digital support tool which is tailored to individual responses to a questionnaire. The tool has been designed for people who are concerned about their drinking. In order to evaluate if the tool is effective, we are asking people who search online to be part of this study.

This research project is conducted at Linköping university in Sweden, please find contact details for the primary investigator below.

What is expected of me? If you take part in the study you will first be asked to respond to a 5 minute questionnaire. Thereafter you will be asked to leave an email address to which we can send more information about the study. You will be randomly assigned to either get direct access to the tool or wait one month before being given access. One month after having signed up for the study we will send an email to you with a link to a short follow-up questionnaire.

Will I be taking any risks by participating in this study? Participating in this study will give you access to a novel digital support tool which has been designed to help people concerned about their drinking. However, you may find that the support given to you is inadequate and does not help you, and you may be de-motivated by this. You should be aware of this before deciding to take part in this study. We are however not asking you to refrain from any other support which may be available to you. For instance, you may find the information available at this website helpful: Rethinking Drinking (https://www.rethinkingdrinking.niaaa.nih.gov/).

Will I be leaving any personal information? We will collect data from you about your age, biological sex, alcohol consumption, and your readiness to change. We will also collect an email address from you so that we can contact you for follow-up one month after enrolling. The data collection is for scientific research, and is therefore motivated by public interest (GDPR EU 2016/679, Prop. 2017/18:298). Your data will be treated with confidentiality and we will take measures to ensure that no unauthorized access is granted.

Linköping University will be responsible for your personal data. According to EU law, you retain the right to, without cost, be given access to your personal data and request that changes be made. You can also request that your personal data be deleted. Your rights for deletion of data is however restricted if the data is deemed necessary for the study to be conducted. If you want access to your personal data you should contact the primary investigator Marcus Bendtsen (see information below). The data protection officer can be contacted at dataskyddsombud@liu.se. If you are not satisfied with how your personal data has been handled, you can make a formal complain to the Swedish Authority for Privacy Protection.

Where will data be stored and who will have access? During the study period we will store all data on a database at Linköping university in Sweden. After the study period the data will be archived securely at Linköping university and removed from the database. Access to the data will at first be restricted to the research team, but will later be made available for other researchers by request. Please note that once the study period is complete, all email addresses will be deleted and data will therefore be anonymous.

Who is responsible for the study? Linköping university is responsible for the data collected in this study. If you have any questions about your data you can contact Dr. Marcus Bendtsen, (marcus.bendtsen@liu.se), or the data protection officer (dataskyddsombudet@liu.se). If you are not satisfied with the way your personal data has been handled, you can file a complaint at the Swedish Data Protection Authority.

How can I get more information about the results from this study? All results will be published in peer-reviewed journals and conferences no later than 2022.

Participation is voluntary. You can at any point decide to not be part of the study and you will not have to explain why.

For peer review only

Contact details
Dr. Marcus Bendtsen, marcus.bendtsen@liu.se, 013-28 69 75
Docent in Medical Informatics
Senior Lecturer in Experimental Social Medicine and Public Health
Department of Health, Medicine and Caring Sciences, Linköping University
581 83 Linköping, Sweden

For peer review only

APPENDIX B - QUESTIONNAIRES

BASELINE AND FOLLOW-UP

1. (Baseline only) How old are you? (numeric)

2. (Baseline only) To assess risks from alcohol consumption, we need to know your biological sex?
    a. Female
    b. Male

3. In the past month, how often did you have six or more drinks on one occasion? (numeric)

4. Thinking about the past week, how many standard drinks did you have on: (numeric)
    a. Monday
    b. Tuesday
    c. Wednesday
    d. Thursday
    e. Friday
    f. Saturday
    g. Sunday

Note: A visual guide will be presented to participants with the definition of a standard drink.

The following questions are designed to identify how you personally feel about your drinking right now. Please think about your current situation and drinking habits, even if you have given up drinking completely. Read each question below carefully and then decide whether you agree or disagree with the statements.

Note: The options for each question below are Strongly disagree, Disagree, Unsure, Agree, Strongly agree. The letter in the parenthesis after the question indicates which stage of change the item belongs to and will not be shown to participants (PC = Precontemplation, C = Contemplation, A = Action).

5. It is a waste of time thinking about my drinking because I do not have a problem. (PC)
6. I enjoy my drinking but sometimes I drink too much. (C)
7. There is nothing seriously wrong with my drinking. (PC)
8. Sometimes I think I should quit or cut down my drinking. (C)
9. Anyone can talk about wanting to do something about their drinking, but I'm actually doing something about it. (A)
10. I am a fairly normal drinker. (PC)
11. My drinking is a problem sometimes. (C)
12. I am actually changing my drinking habits right now (either cutting down or quitting). (A)
13. I have started to carry out a plan to cut down or quit drinking. (A)
14. There is nothing I really need to change about my drinking. (PC)
15. Sometimes I wonder if my drinking is out of control. (C)
16. I am actively working on my drinking problem. (C)

For peer review only

APPENDIX C – DEBRIEFING EMAIL

The following email will be sent to the waiting list group after study completion.

Thank you for taking part in the "Digital support for alcohol" study.

The point of the study was to explore if being told to wait for the alcohol support tool made a difference to your alcohol consumption. Therefore, the material you received when you signed up for the study was the support tool which the other group also received. They were however told that they were given the support tool immediately.

This research is important because many studies use a design where participants are asked to wait before they get a new treatment, and it is uncertain what the effects of being told to wait are.

If you have questions about the study and its design, you are welcome to contact the primary investigator Marcus Bendtsen (see below for details).

If you want more support for change of your alcohol consumption you may find the information on this website helpful: Rethinking Drinking (https://www.rethinkingdrinking.niaaa.nih.gov/).

Contact details
Dr. Marcus Bendtsen, marcus.bendtsen@liu.se, 013-28 69 75
Docent in Medical Informatics
Senior Lecturer in Experimental Social Medicine and Public Health
Department of Health, Medicine and Caring Sciences, Linköping University
581 83 Linköping, Sweden

For peer review only

SPIRIT 2013 Checklist: Recommended items to address in a clinical trial protocol and related documents*

Section/item	Item No	Description	Page Number on which item is reported
**Administrative information**			
Title	1	Descriptive title identifying the study design, population, interventions, and, if applicable, trial acronym	1
Trial registration	2a	Trial identifier and registry name. If not yet registered, name of intended registry	2
	2b	All items from the World Health Organization Trial Registration Data Set	2
Protocol version	3	Date and version identifier	NA
Funding	4	Sources and types of financial, material, and other support	2,12
Roles and responsibilities	5a	Names, affiliations, and roles of protocol contributors	1
	5b	Name and contact information for the trial sponsor	NA
	5c	Role of study sponsor and funders, if any, in study design; collection, management, analysis, and interpretation of data; writing of the report; and the decision to submit the report for publication, including whether they will have ultimate authority over any of these activities	NA
	5d	Composition, roles, and responsibilities of the coordinating centre, steering committee, endpoint adjudication committee, data management team, and other individuals or groups overseeing the trial, if applicable (see Item 21a for data monitoring committee)	NA
**Introduction**			

For peer review only - http://bmjopen.bmj.com/site/about/guidelines.xhtml

Background and rationale	6a	Description of research question and justification for undertaking the trial, including summary of relevant studies (published and unpublished) examining benefits and harms for each intervention	3-4
	6b	Explanation for choice of comparators	3-4
Objectives	7	Specific objectives or hypotheses	4
Trial design	8	Description of trial design including type of trial (eg, parallel group, crossover, factorial, single group), allocation ratio, and framework (eg, superiority, equivalence, noninferiority, exploratory)	4
**Methods: Participants, interventions, and outcomes**			
Study setting	9	Description of study settings (eg, community clinic, academic hospital) and list of countries where data will be collected. Reference to where list of study sites can be obtained	4-5
Eligibility criteria	10	Inclusion and exclusion criteria for participants. If applicable, eligibility criteria for study centres and individuals who will perform the interventions (eg, surgeons, psychotherapists)	4-5
Interventions	11a	Interventions for each group with sufficient detail to allow replication, including how and when they will be administered	5
	11b	Criteria for discontinuing or modifying allocated interventions for a given trial participant (eg, drug dose change in response to harms, participant request, or improving/worsening disease)	NA
	11c	Strategies to improve adherence to intervention protocols, and any procedures for monitoring adherence (eg, drug tablet return, laboratory tests)	4-5
	11d	Relevant concomitant care and interventions that are permitted or prohibited during the trial	NA
Outcomes	12	Primary, secondary, and other outcomes, including the specific measurement variable (eg, systolic blood pressure), analysis metric (eg, change from baseline, final value, time to event), method of aggregation (eg, median, proportion), and time point for each outcome. Explanation of the clinical relevance of chosen efficacy and harm outcomes is strongly recommended	6

For peer review only - http://bmjopen.bmj.com/site/about/guidelines.xhtml

Participant timeline	13	Time schedule of enrolment, interventions (including any run-ins and washouts), assessments, and visits for participants. A schematic diagram is highly recommended (see Figure)	Figure 2
Sample size	14	Estimated number of participants needed to achieve study objectives and how it was determined, including clinical and statistical assumptions supporting any sample size calculations	8
Recruitment	15	Strategies for achieving adequate participant enrolment to reach target sample size	4,8
**Methods: Assignment of interventions (for controlled trials)**			
Allocation:			
Sequence generation	16a	Method of generating the allocation sequence (eg, computer-generated random numbers), and list of any factors for stratification. To reduce predictability of a random sequence, details of any planned restriction (eg, blocking) should be provided in a separate document that is unavailable to those who enrol participants or assign interventions	7
Allocation concealment mechanism	16b	Mechanism of implementing the allocation sequence (eg, central telephone; sequentially numbered, opaque, sealed envelopes), describing any steps to conceal the sequence until interventions are assigned	7
Implementation	16c	Who will generate the allocation sequence, who will enrol participants, and who will assign participants to interventions	7
Blinding (masking)	17a	Who will be blinded after assignment to interventions (eg, trial participants, care providers, outcome assessors, data analysts), and how	7
	17b	If blinded, circumstances under which unblinding is permissible, and procedure for revealing a participant's allocated intervention during the trial	NA
**Methods: Data collection, management, and analysis**			

Data collection methods	18a	Plans for assessment and collection of outcome, baseline, and other trial data, including any related processes to promote data quality (eg, duplicate measurements, training of assessors) and a description of study instruments (eg, questionnaires, laboratory tests) along with their reliability and validity, if known. Reference to where data collection forms can be found, if not in the protocol	6
	18b	Plans to promote participant retention and complete follow-up, including list of any outcome data to be collected for participants who discontinue or deviate from intervention protocols	9
Data management	19	Plans for data entry, coding, security, and storage, including any related processes to promote data quality (eg, double data entry; range checks for data values). Reference to where details of data management procedures can be found, if not in the protocol	NA
Statistical methods	20a	Statistical methods for analysing primary and secondary outcomes. Reference to where other details of the statistical analysis plan can be found, if not in the protocol	7-8
	20b	Methods for any additional analyses (eg, subgroup and adjusted analyses)	7-8
	20c	Definition of analysis population relating to protocol non-adherence (eg, as randomised analysis), and any statistical methods to handle missing data (eg, multiple imputation)	7-8
**Methods: Monitoring**			
Data monitoring	21a	Composition of data monitoring committee (DMC); summary of its role and reporting structure; statement of whether it is independent from the sponsor and competing interests; and reference to where further details about its charter can be found, if not in the protocol. Alternatively, an explanation of why a DMC is not needed	NA
	21b	Description of any interim analyses and stopping guidelines, including who will have access to these interim results and make the final decision to terminate the trial	8

For peer review only - http://bmjopen.bmj.com/site/about/guidelines.xhtml

Harms	22	Plans for collecting, assessing, reporting, and managing solicited and spontaneously reported adverse events and other unintended effects of trial interventions or trial conduct	NA
Auditing	23	Frequency and procedures for auditing trial conduct, if any, and whether the process will be independent from investigators and the sponsor	NA
**Ethics and dissemination**			
Research ethics approval	24	Plans for seeking research ethics committee/institutional review board (REC/IRB) approval	9
Protocol amendments	25	Plans for communicating important protocol modifications (eg, changes to eligibility criteria, outcomes, analyses) to relevant parties (eg, investigators, REC/IRBs, trial participants, trial registries, journals, regulators)	NA
Consent or assent	26a	Who will obtain informed consent or assent from potential trial participants or authorised surrogates, and how (see Item 32)	4-5
	26b	Additional consent provisions for collection and use of participant data and biological specimens in ancillary studies, if applicable	NA
Confidentiality	27	How personal information about potential and enrolled participants will be collected, shared, and maintained in order to protect confidentiality before, during, and after the trial	NA
Declaration of interests	28	Financial and other competing interests for principal investigators for the overall trial and each study site	12
Access to data	29	Statement of who will have access to the final trial dataset, and disclosure of contractual agreements that limit such access for investigators	9
Ancillary and post-trial care	30	Provisions, if any, for ancillary and post-trial care, and for compensation to those who suffer harm from trial participation	NA
Dissemination policy	31a	Plans for investigators and sponsor to communicate trial results to participants, healthcare professionals, the public, and other relevant groups (eg, via publication, reporting in results databases, or other data sharing arrangements), including any publication restrictions	9

For peer review only

	31b	Authorship eligibility guidelines and any intended use of professional writers	NA
	31c	Plans, if any, for granting public access to the full protocol, participant-level dataset, and statistical code	9
**Appendices**			
Informed consent materials	32	Model consent form and other related documentation given to participants and authorised surrogates	Appendix A
Biological specimens	33	Plans for collection, laboratory evaluation, and storage of biological specimens for genetic or molecular analysis in the current trial and for future use in ancillary studies, if applicable	NA

*It is strongly recommended that this checklist be read in conjunction with the SPIRIT 2013 Explanation & Elaboration for important clarification on the items. Amendments to the protocol should be tracked and dated. The SPIRIT checklist is copyrighted by the SPIRIT Group under the Creative Commons "Attribution-NonCommercial-NoDerivs 3.0 Unported" license.

For peer review only
